# Multiomic profiling reveals metabolic alterations mediating aberrant platelet activity and inflammation in myeloproliferative neoplasms

**DOI:** 10.1172/JCI172256

**Published:** 2024-02-01

**Authors:** Fan He, Angelo B.A. Laranjeira, Tim Kong, Shuyang Lin, Katrina J. Ashworth, Alice Liu, Nina M. Lasky, Daniel A.C. Fisher, Maggie J. Cox, Mary C. Fulbright, Lilian Antunes-Heck, LaYow Yu, Molly Brakhane, Bei Gao, Stephen M. Sykes, Angelo D’Alessandro, Jorge Di Paola, Stephen T. Oh

**Affiliations:** 1 Division of Hematology, Department of Medicine, and; 2 Division of Hematology & Oncology, Department of Pediatrics, School of Medicine, Washington University School of Medicine, St. Louis, Missouri, USA.; 3 Department of Biochemistry and Molecular Genetics, University of Colorado-Anschutz Medical Campus, Aurora, Colorado, USA.; 4 Immunomonitoring Laboratory, Center for Human Immunology and Immunotherapy Programs, and; 5 Department of Pathology and Immunology, Washington University School of Medicine, St. Louis, Missouri, USA.

**Keywords:** Hematology, Platelets

## Abstract

Platelets from patients with myeloproliferative neoplasms (MPNs) exhibit a hyperreactive phenotype. Here, we found elevated P-selectin exposure and platelet-leukocyte aggregates indicating activation of platelets from essential thrombocythemia (ET) patients. Single-cell RNA-seq analysis of primary samples revealed significant enrichment of transcripts related to platelet activation, mTOR, and oxidative phosphorylation in ET patient platelets. These observations were validated via proteomic profiling. Platelet metabolomics revealed distinct metabolic phenotypes consisting of elevated ATP generation accompanied by increases in the levels of multiple intermediates of the tricarboxylic acid cycle, but lower α-ketoglutarate (α-KG) in MPN patients. Inhibition of PI3K/AKT/mTOR signaling significantly reduced metabolic responses and hyperreactivity in MPN patient platelets, while α-KG supplementation markedly reduced oxygen consumption and ATP generation. Ex vivo incubation of platelets from both MPN patients and *Jak2* *V617F*–knockin mice with α-KG supplementation significantly reduced platelet activation responses. Oral α-KG supplementation of *Jak2* *V617F* mice decreased splenomegaly and reduced hematocrit, monocyte, and platelet counts. Finally, α-KG treatment significantly decreased proinflammatory cytokine secretion from MPN CD14^+^ monocytes. Our results reveal a previously unrecognized metabolic disorder in conjunction with aberrant PI3K/AKT/mTOR signaling that contributes to platelet hyperreactivity in MPN patients.

## Abstract 2

<p>Multiomic profiling from patients with myeloproliferative neoplasms reveals aberrant platelet metabolism, activity, and inflammation that is corrected in mice by &alpha;-KG supplementation.</p>

## Introduction

Philadelphia-negative chronic myeloproliferative neoplasms (MPNs), including polycythemia vera (PV), essential thrombocythemia (ET), and myelofibrosis (MF), are clonal hematopoietic disorders characterized by overproduction of mature blood cells such as erythrocytes, granulocytes, and/or platelets (1). MPN patients have a significantly elevated risk of thrombosis, with an estimated pooled prevalence of overall thrombosis among patients with MPN of 20% at diagnosis (2). Cytoreductive therapies and aspirin are commonly used for the treatment of MPN patients to reduce thrombosis risk (3).

MPN-associated thrombosis is considered a multifactorial event involving the complex interplay of blood and endothelial cells, the coagulation cascade, *JAK2* mutation allele burden, and chronic inflammation, all of which likely contribute to the prothrombotic phenotype (4). Increased thrombin generation and elevated levels of procoagulant microparticles and soluble P-selectin are seen in MPN patients and suggest hyperreactivity of platelets (5–7). Platelets from homozygous *JAK2* *V617F*–knockin mice, which exhibit a PV-like phenotype, have reduced aggregation and increased bleeding time, whereas heterozygous *JAK2* *V617F* mice, which exhibit an ET-like phenotype, showed increased platelet aggregation and reduced bleeding time (8, 9). Chronic inflammation is a hallmark of MPNs regardless of subtype (10). Aside from its classical functions in hemostasis, platelets also contribute to thromboinflammatory processes by directly interacting with leukocytes (11). Elevated platelet-leukocyte aggregates (PLAs) have recently been found to be an independent risk factor for MPN-associated thrombosis, indicating that platelets could be a mediator between thrombosis and inflammation (12–14). While it is known that thrombotic risk in MPNs is not directly correlated with the platelet count, the factors mediating aberrant activation of platelets in MPNs have not been systematically evaluated.

Human platelets contain mitochondria, which supply approximately 40% of the energy during the resting state. Oxidative phosphorylation (OXPHOS) increases significantly during platelet activation and secretion to meet the increased demand for energy (15). The dysregulation of platelet metabolism has been reported in several diseases, such as diabetes mellitus, sepsis, and pulmonary hypertension (15). Previously, we reported that tumor necrosis factor α (TNF-α) induces mitochondrial dysfunction in mice, which was associated with platelet hyperreactivity, particularly during aging (16). We also reported increased mitochondrial mass and platelet hyperreactivity in platelets from patients with MPNs, a disease associated with chronic inflammation and significantly high levels of TNF-α (10, 16–18). However, despite this clear association, the mechanisms driving metabolic dysregulation in MPN patient platelets remain incompletely understood.

Here, we apply multiomic approaches in conjunction with functional interrogation of platelets from MPN patients to delineate mechanisms of platelet hyperreactivity. We confirm that MPN patient platelets exhibit increased activation and uncover metabolic alterations, such as in OXPHOS and mTOR signaling activation, as critical contributors to this platelet hyperreactivity. In addition, across MPN primary samples and mouse models, we demonstrate that α-ketoglutarate (α-KG) supplementation suppresses platelet activation and megakaryopoiesis, in part due to inhibition of cellular metabolism and mTOR pathway signaling, thereby identifying a promising therapeutic strategy for MPN patients.

## Results

### ET patients show significantly increased P-selectin levels and PLA formation.

**Figure 1:**
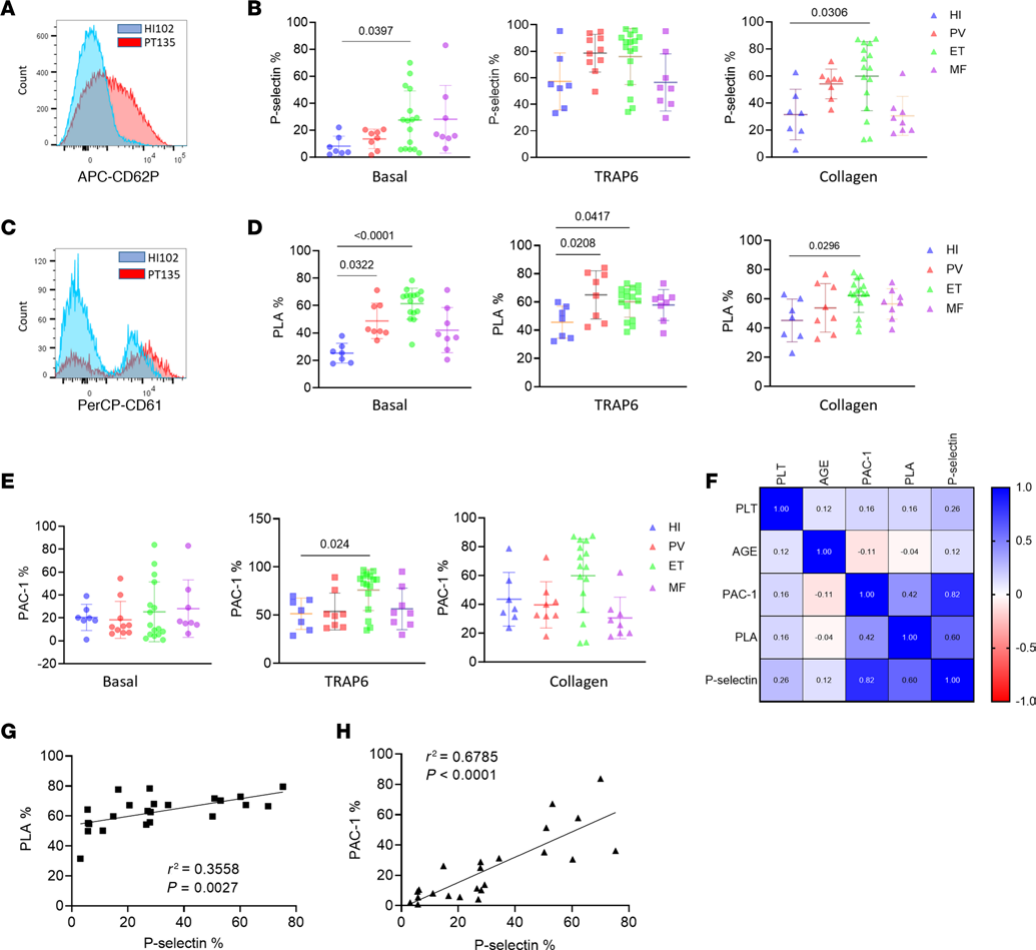
Platelets from ET patients show significantly increased P-selectin level and PLA formation. (**A**) Representative figure of exposure of P-selectin on the surface of platelets measured by flow cytometry. (**B**) P-selectin expression on the surface of platelets at baseline and following 1 μM TRAP6 or 5 μg/mL collagen stimulation (HI = 7, PV = 8, ET = 16, MF = 8). Data are mean ± SD and were assessed by Kruskal-Wallis test. (**C**) Representative figure of PLA ratio measured by flow cytometry. Data are presented as percentages of aggregates from the respective leukocyte population. (**D**) PLA measurements in whole blood at baseline and following 1 μM TRAP6 or 5 μg/mL collagen stimulation (HI = 7, PV =8, ET = 16, MF = 8). Data are mean ± SD and were assessed by Kruskal-Wallis test. (**E**) αIIbβ3 integrin expression (presented as the percentage positive staining of anti–PAC-1 antibody; see Supplemental Table 2) at baseline and following 1 μM TRAP6 or 5 μg/mL collagen stimulation (HI = 7, PV = 8, ET = 16, MF = 8). Data are mean ± SD and were assessed by Brown-Forsythe and Welch’s ANOVA test. (**F**) Pearson’s correlation coefficient among platelet (PLT) markers and parameters in ET. (**G**) Simple linear regression between PLA percentage and P-selectin percentage in ET. (**H**) Simple linear regression between αIIbβ3 integrin percentage and P-selectin percentage in ET.

First, platelet activation was measured in peripheral blood from 32 MPN (PV = 8, ET = 16, MF = 8) patients and healthy individuals (HIs, *n* = 7; Table 1). There were no significant differences in age and sex between MPN patients and HIs. Platelets from ET patients had significantly higher basal P-selectin, a marker of α-granule activation and secretion, compared with HIs, with no significant differences observed after platelet agonist thrombin receptor activator peptide 6 (TRAP6) stimulation (Figure 1, A and B). We also observed increased PLA formation in PV and ET peripheral blood samples (Figure 1, C and D), consistent with previous reports (19). Platelets from ET patients had higher αIIbβ3 integrin activation following TRAP6 stimulation, but there was no difference at baseline (Figure 1E). P-selectin exposure positively correlated with αIIbβ3 integrin activation and PLA formation, as expected (Figure 1, F–H, and Table 2).

Platelets from ET patients carrying *JAK2* mutations exhibited stronger responses to TRAP6 stimulation than those with *CALR* mutations, as indicated by higher P-selectin exposure and αIIbβ3 integrin activation, which is consistent with the lower risk of thrombosis reported in *CALR*-mutant patients compared with *JAK2* (Table 3 and Supplemental Figure 1A; supplemental material available online with this article; https://doi.org/10.1172/JCI172256DS1) (20). Platelet aggregometry showed decreased platelet responses in MPN patients when compared with HIs, likely related to the effects of aspirin (Supplemental Figure 1, B and C). However, platelet aggregation responses were significantly higher in patients with the *JAK2* mutation when compared with those carrying *CALR* mutations (Supplemental Figure 1D). Overall, our results show elevated P-selectin levels and increased αIIbβ3 integrin activation and PLA formation, indicating hyperreactivity of platelets in patients with MPNs. To interrogate the significance of PLA formation, we coincubated platelets and monocytes and measured cytokine production by monocytes. Boosted secretion of proinflammatory cytokines by monocytes in the presence of platelets suggested that PLA increases in MPN patients might also contribute to hyperinflammation (Supplemental Figure 1E).

### Platelets from ET patients show enrichment of genes involved in platelet activation, PI3K/AKT/mTOR signaling, and OXPHOS.

**Figure 2:**
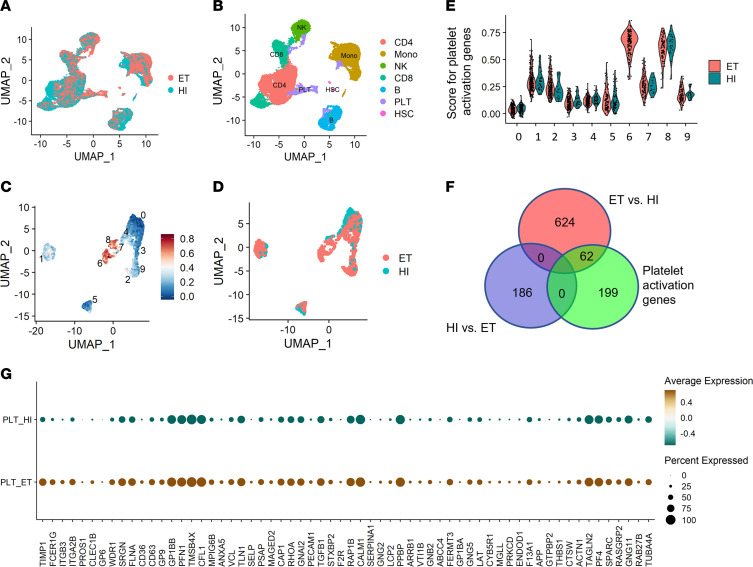
scRNA-seq revealed the activation of platelets and monocytes in peripheral blood from ET patients. (**A**) UMAP plot of cells sequenced from HIs (*n* = 3) and ET patients (*n* = 5). (**B**) UMAP plot of cells sequenced from HIs and ET patients with cell type annotations. PLT, platelet; HSC, hematopoietic stem cell. (**C**) UMAP plot showing platelets clustering with scores for “reactome platelet activation signaling and aggregation” gene set. (**D**) UMAP plot showing platelets from HIs and ET. (**E**) Violin plot of platelet clusters showing scores for “reactome platelet activation signaling and aggregation” gene set. (**F**) Venn diagram showing overlapped genes among differentially expressed genes in platelets from HIs, ET patients, and genes in “reactome platelet activation signaling and aggregation” gene set. (**G**) Dot plot of genes in “reactome platelet activation signaling and aggregation” gene set that overlapped with differentially expressed genes in platelets from HIs (0/186) and ET patients (62/686).

Based on our findings of increased platelet reactivity and PLA formation, and to better understand the transcriptional landscape of platelets and other blood cells from MPN patients, we performed single-cell RNA-seq (scRNA-seq) of peripheral blood samples from ET patients (*n* = 5) and age- and sex-matched HIs (*n* = 3) (Figure 2A and Table 4). Cell types were assigned according to their canonical transcripts, such as *CD14* for CD14^+^ monocytes, *CD8A* for CD8^+^ T cells, and *PPBP* for platelets (Figure 2B and Supplemental Figure 2A). Cell clusters showed distinct transcriptional enrichments (Supplemental Figure 2B). Subsequent cell composition analysis showed that CD4^+^ T cells were the most abundant cell type in both ET patients and HIs, accounting for 30%–55% of cells (Supplemental Figure 2C). Percentages of platelets and monocytes increased in ET patients (Supplemental Figure 2C). Platelets were further clustered into 10 distinct groups using unsupervised clustering methods (Figure 2, C and D). Platelets in cluster 6 exhibited the highest expression score for the platelet activation gene set and were all from ET patients, whereas cluster 0 with the lowest expression score was predominantly represented by HI platelets (Figure 2, C and E). We then interrogated the overlap of the platelet activation gene set (composed of 261 genes) with differentially expressed genes in platelets from HIs and ET patients. While none of the 261 genes were enriched in platelets from HIs, 62 genes were upregulated in platelets from ET patients, including *SELP*, *PF4*, and *GP1BA* (Figure 2, F and G). Consistently, unbiased Gene Ontology (GO) and Kyoto Encyclopedia of Genes and Genomes (KEGG) analyses showed enrichment of genes involved in platelet activation in ET patients (Supplemental Figure 2, D–F). Gene set enrichment analysis (GSEA) confirmed increases in transcripts associated with platelet activation and IFN-γ pathways, and showed upregulation of genes involved in PI3K/AKT/mTOR signaling and OXPHOS in ET platelets (Supplemental Figure 2, G–K). Of note, IFN and TNF signaling pathways were further enriched in platelets from 3 *JAK2*-mutant patients compared with 2 *CALR*-mutant patients (Supplemental Figure 2H), which might be relevant to the lower platelet activation we observed above and decreased thrombosis risk reported previously in *CALR*-mutant MPNs (21). We also performed transcription factor (TF) analysis using DoRothEA, a curated collection of TFs and their transcriptional targets (22). The enrichment of specific TFs in blood cells, such as *GATA1* in platelets and *ZEB2* in T cells, is consistent with previous studies (23, 24), which in turn validated the accuracy of our cell identity assignments (Supplemental Figure 2, L and M). In addition, we observed upregulation of *GATA1* and *STAT1*, important regulators of megakaryopoiesis, in platelets from ET patients, which echoes elevated platelet counts observed in ET patients (Supplemental Figure 2, N and O) (25).

To validate our scRNA-seq findings, we analyzed GSE2006 (26), a publicly available comparative microarray of platelets from ET patients and HIs, and found enrichment of platelet activation and OXPHOS gene sets in platelets from ET patients (Supplemental Figure 3, A–D). We also observed significant upregulation of representative platelet activation genes in GSE2006, including *TIMP1*, *ITGB3*, *ITGA2B*, *FLNA*, *GP6*, *SELP*, and *CD36* (fold change > 2, *P* < 0.05; Supplemental Figure 3E), replicating our scRNA-seq results. Thus, these results not only support the hyperactivation of platelets in MPN patients, but also led to the hypothesis that platelet metabolic changes contribute to the platelet hyperreactivity observed in MPNs.

Gene expression analyses of IFN-γ and inflammation response pathways revealed that monocytes exhibited the highest score of all blood cells analyzed (Supplemental Figure 4, A and C). Notably, monocytes from patients with *JAK2* *V617F* showed higher activities in IFN-γ and inflammation response pathways compared with patients with *CALR* mutations (Supplemental Figure 4, B and D). Enrichments of SPI1 and IRF TFs in monocytes from ET patients support our observations (Supplemental Figure 4E). Further clustering showed that 2 distinct monocyte clusters (8 and 9) exhibited the highest transcriptional levels of IFN-γ pathway genes (Supplemental Figure 4, F and G). Of note, these 2 clusters also showed robust enrichments of monocytes from ET patients and higher levels of *PPBP*, a platelet marker, suggesting a role of platelets in activating monocytes (Supplemental Figure 4, H and I). These observations supported the results of our platelet-monocyte coincubation experiments (Supplemental Figure 1E). GSEA showed enrichment of IFN signaling and metabolism pathways in monocytes from ET patients (Supplemental Figure 4J). Thus, monocytes from ET patients display elevated inflammation, with increased PLA formation potential contributing.

### Proteomics and metabolomics analyses confirm elevated PI3K/AKT/mTOR signaling and mitochondrial activity in MPN platelets.

**Figure 3:**
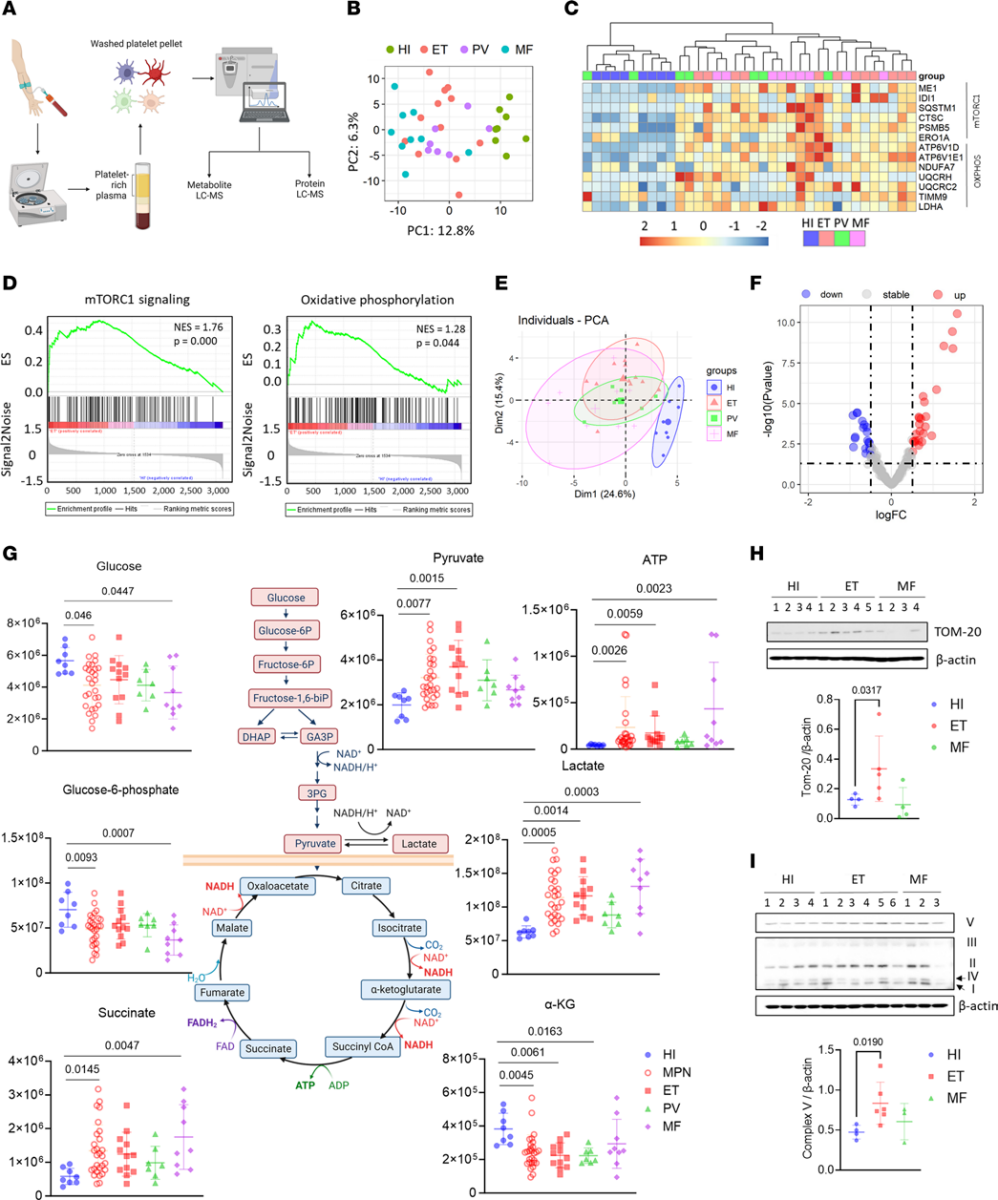
Metabolomics analyses showed distinct metabolic phenotypes of platelets from MPN patients. (**A**) Diagram showing sample collection, processing, and analysis. (**B**) PCA score plot of top 500 most variable proteins in protein LC-MS data of platelets from age- and sex-matched HIs (*n* = 8) and MPN patients (ET = 12, PV = 7, MF = 9). (**C**) Heatmap of selected proteins from “hallmark mTORC1 signaling” and “hallmark OXPHOS” gene sets. Columns were reordered based on the results of hierarchical clustering to identify sample correlations. (**D**) GSEA enrichment plots for “hallmark mTORC1 signaling” and “hallmark OXPHOS” gene sets enriched in ET patients versus HIs. (**E**) Principal component analysis (PCA) score plot of metabolite LC-MS data of platelets from age- and sex-matched HIs (*n* = 8) and MPN patients (ET = 12, PV = 7, MF = 9) displayed with 80% confidence region. (**F**) Volcano plot of metabolite changes between HIs and MPN patients. Red dots denote significant (*P* < 0.05) and positive fold change (logFC > 2^0.5^) features. Blue dots denote significant (*P* < 0.05) and negative fold change (logFC < –2^0.5^) features. (**G**) The diagram showing steps of glycolysis and TCA cycle and scatter plots of peak areas (arbitrary units after normalization) for several key metabolites. Data are mean ± SD and were assessed by Kruskal-Wallis test with Dunn’s multiple-comparison test. *P* values are marked if less than 0.05. (**H**) Western blot of washed platelets from HIs and MPN patients against TOM-20 (see Supplemental Table 2), a mitochondrial marker protein, and quantifications. Data are mean ± SD and were assessed by 2-tailed Mann-Whitney *U* test. (**I**) Western blot of washed platelets from HIs and MPN patients detecting human OXPHOS components (complex I–V proteins) with an antibody cocktail and quantifications. Data are mean ± SD and were assessed by 2-tailed Mann-Whitney *U* test.

To better understand metabolic alterations in MPN platelets, we performed ultra-high-performance liquid chromatography coupled with mass spectrometry (LC-MS) metabolomics on paired plasma and washed platelets from MPN patients along with age- and sex-matched HIs (Figure 3A and Table 5; ET = 12, PV = 7, MF = 9, HI = 8). Platelet proteomics showed evident clustering of MPN samples in the PCA plot (Figure 3B). Enrichment of genes involved in mTOR signaling and OXPHOS pathways in platelets from MPN patients was also observed in proteomics analyses (Figure 3, C and D), consistent with our scRNA-seq data. Further validating our proteomics results, MPN platelets showed significantly elevated RNA and protein levels of ME1 and CTSC, mTORC1 signaling proteins, compared with HIs (Supplemental Figure 5, A and B).

For metabolomics analysis, we first performed a multivariate analysis to reduce dimensionality for visualization, which revealed a clear distinction between HI and MPN platelets (Figure 3E). Differentially accumulated metabolites (DAMs) were defined as those exhibiting a |log(fold change)| greater than 0.5 and *P* less than 0.05 between MPN patients and HIs. In comparing MPN and HIs, 24 of 181 (13.3%) were upregulated and 19 of 181 (10.5%) were downregulated; 6-phosphogluconate, α-KG, succinate, and ATP were among the top DAMs (Figure 3F and Supplemental Figure 5C). In contrast, the lack of differences in plasma metabolites between HIs and MPN patients suggested that the metabolic changes in platelets are intrinsic modifications instead of changes in the microenvironment (Supplemental Figure 5D). Lower glucose in conjunction with higher pyruvate levels indicate activation of glycolytic pathways in platelets from MPN patients compared with HIs (Figure 3G). Increased Krebs cycle components paralleled higher ATP levels, suggesting elevated mitochondrial activities in MPN patient platelets (Figure 3G). Measurement of mitochondrial protein and OXPHOS complexes by immunoblotting demonstrated significant increases in TOM-20, a mitochondrial marker, and complex V protein, the ATP synthase responsible for energy generation, in platelets from ET patients (Figure 3, H and I). Overall, our results revealed elevated OXPHOS and mitochondrial activities in MPN platelets.

Metabolomics analysis revealed additional changes in MPN platelets. Significantly increased levels of lactate in MPN platelets echoes a previous report describing the alteration of glycolysis in MPN (27). We also found increases in several amino acids in MPN platelets, including proline, glutamate, and glutamine, suggesting enhanced protein synthesis (Supplemental Figure 5E). Increases in *S*-adenosylmethionine (SAM), the methyl donor in cytosine methylation, and methionine suggest hypermethylation in MPN platelets (Supplemental Figure 5F). We further compared metabolite changes among ET patients carrying different mutations (4 *CALR* and 8 *JAK2*), with no major differences identified via PCA (Supplemental Figure 5G). DAMs included only 3 upregulated (tetradecanoic acid, tetradecenoic acid, and hexadecenoic acid) and 4 downregulated (L-cysteine, glutathione, 2,3-phospho-D-glycerate, and UTP) metabolites in platelets from *JAK2*-mutant ET patients (Supplemental Figure 5H). Thus, we did not observe substantial metabolic changes in platelets among MPN patients carrying *JAK2* or *CALR* mutations. Processed metabolomics data are provided in Supplemental Table 1.

### Inhibition of PI3K/AKT/mTOR signaling restrains MPN platelet hyperactivation.

**Figure 4:**
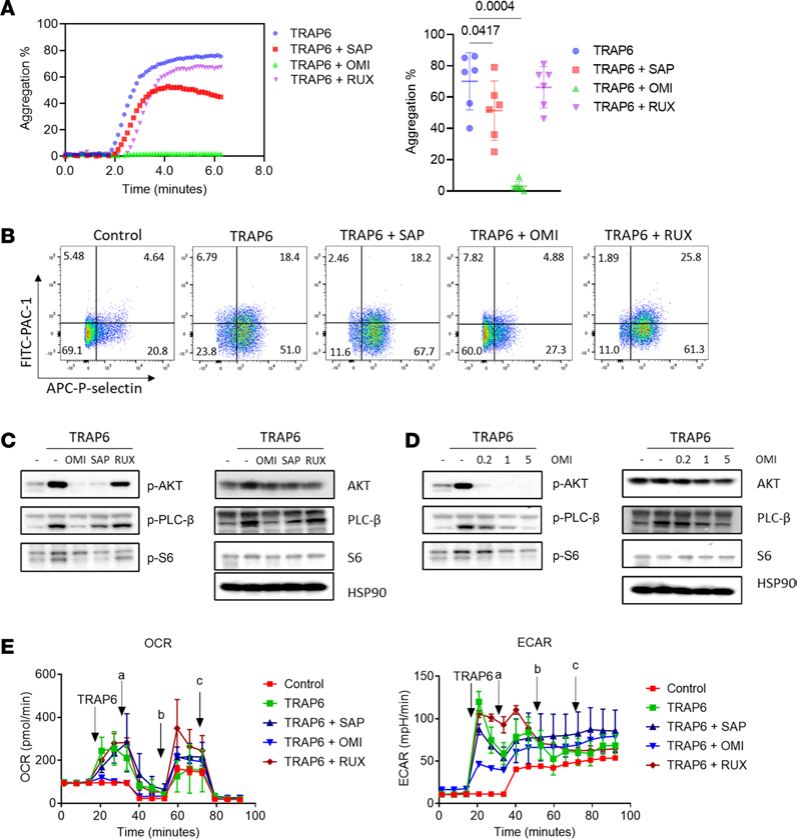
Effects of mTOR inhibitors and ruxolitinib on platelet activities. (**A**) Representative image and dot plot showing effects of mTOR inhibitors and ruxolitinib on maximal aggregation intensity of washed ET platelets. Washed platelets were treated with sapanisertib, omipalisib, or ruxolitinib at 5 μM for 1 hour followed by platelet aggregation analysis with 5 μM TRAP6 stimulation. Data shown as mean ± SD and were assessed by Friedman’s test and Dunn’s multiple-comparison test. (**B**) Representative images showing effects of mTOR inhibitors and ruxolitinib on activation of washed ET platelets. Washed platelets were treated with sapanisertib, omipalisib, or ruxolitinib at 5 μM for 1 hour followed by flow cytometry analysis. (**C**) Immunoblots showing changes in intracellular signaling pathways of platelets after mTOR inhibitor and ruxolitinib treatments. Washed platelets were treated with sapanisertib, omipalisib, or ruxolitinib at 5 μM for 1 hour followed by stimulation with TRAP6 peptides and immunoblot analysis. (**D**) Immunoblots showing changes in intracellular signaling pathways of MPN platelets after omipalisib treatment. Washed platelets were treated with omipalisib at 0.2, 1, and 5 μM for 1 hour followed by stimulation with TRAP6 peptides and immunoblot analysis. (**E**) Representative OCR and ECAR profiles of platelets showing the blockage of energy demand boost by mTOR inhibitors after TRAP6 stimulation. Washed ET platelets were treated with sapanisertib, omipalisib, or ruxolitinib at 5 μM for 1 hour followed by Seahorse analysis (a, oligomycin A; b, FCCP; c, rotenone/antimycin A). TRAP6 (20 μM) was injected on-plate to stimulate platelet energy demand.

To investigate the role of PI3K/AKT/mTOR signaling in MPN platelet hyperactivation, we treated washed platelets from MPN patients with mTOR inhibitors. Omipalisib, a dual PI3K/mTOR inhibitor, abrogated all platelet activation and aggregation, whereas a milder, but still significant effect was observed by the PI3K-sparring mTOR inhibitor sapanisertib (Figure 4, A and B). In contrast, ruxolitinib, a JAK2 inhibitor approved for MPN treatment, had no effects on platelet activity. In functional experiments, mTOR inhibitors showed dose-dependent inhibition of platelet aggregation and activation (Supplemental Figure 6, A and B). Apoptosis assays were performed to determine whether the effects of mTOR inhibitors on MPN platelet hyperactivation may be related to induction of apoptosis. No significant increases in phosphatidylserine exposure and cleaved caspase-3 level were observed with omipalisib and sapanisertib incubation (Supplemental Figure 6, C and D). A1331852, a BCL-XL selective inhibitor, induced significant phosphatidylserine exposure and cleaved caspase-3 level as a positive control (Supplemental Figure 6, C and D). Further examination of platelet intracellular signaling pathways by immunoblotting revealed that mTOR inhibitors significantly reduced phosphorylation of AKT and PLC-β induced by TRAP6 stimulation in a dose-dependent manner (Figure 4, C and D). Incubation of omipalisib and sapanisertib inhibited platelet oxygen consumption rate (OCR) and ATP generation without affecting ROS level (Supplemental Figure 6, E–G). Notably, incubation of omipalisib with platelets completely blocked the increase in mitochondrial respiration induced by TRAP6 stimulation, which was not observed with sapanisertib or ruxolitinib (Figure 4E). Taken together, these results highlight key roles played by PI3K/AKT/mTOR signaling activation and elevated mitochondrial activity in MPN platelet hyperactivation.

### MPN platelets display bioenergetic changes that can be reverted by α-KG supplementation.

**Figure 5:**
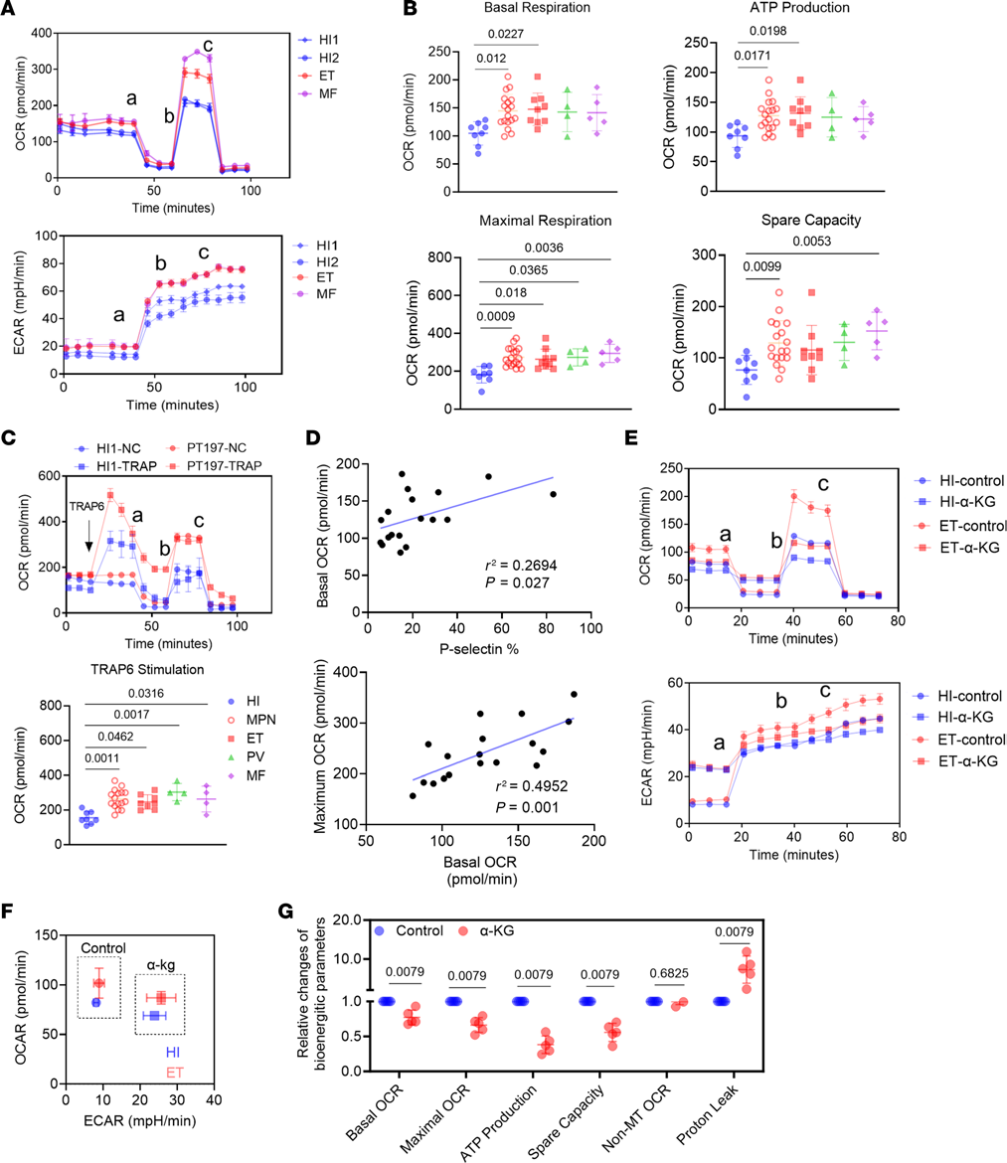
Platelets from MPN patients displayed bioenergetic alterations, which can be reverted by α-KG supplementation. (**A**) Representative OCR and ECAR profiles of platelets from 2 HIs, 1 ET patient, and 1 MF patient (a, oligomycin A; b, FCCP; c, rotenone/antimycin A). (**B**) Quantification of basal OCR, ATP production, maximal OCR, and spare capacity profiles of washed platelets in HIs (*n* = 8) and MPN patients (*n* = 18: ET = 9, PV = 4, MF = 5). Data are mean ± SD and were assessed by Kruskal-Wallis test with Dunn’s multiple-comparison test. *P* values are marked if less than 0.05. (**C**) Representative OCR profiles of platelets from 1 HI and 1 ET patient showing the energy demand boost after TRAP6 stimulation and quantification of post-TRAP6 stimulation OCR profiles (a, oligomycin A; b, FCCP; c, rotenone/antimycin A). Data are mean ± SD and were assessed by Kruskal-Wallis test with Dunn’s multiple-comparison test. *P* values are marked if less than 0.05. (**D**) Correlation analysis of bioenergetic parameters and platelet functional parameters. (**E**) Representative OCR and ECAR profiles of platelets from 1 HI and 1 ET patient with the preincubation of 500 μM octyl-α-KG or DMSO for 1 hour (a, oligomycin A; b, FCCP; c, rotenone/antimycin A). (**F**) Platelet OCR/ECAR ratio from 1 HI and 1 ET patient with the preincubation of 500 μM octyl-α-KG or DMSO control. (**G**) Quantification of individual components of the platelet OCR profile in MPN (*n* = 5). Data were normalized to DMSO group set as 1, are presented as mean ± SD, and were assessed by 2-tailed Mann-Whitney *U* test.

To interrogate bioenergetic changes, MPN platelets were isolated for Seahorse extracellular flux analysis. MPN platelets showed increased rates of basal respiration and ATP generation after correction for non-mitochondrial OCR, which suggested elevated physiological mitochondrial respiration (Figure 5, A and B). Platelets from *Jak2 V617F* mice also showed slightly higher basal respiration and ATP generation when compared with those from wild-type mice (Supplemental Figure 7, A and B). The reserve capacity, calculated as the difference between maximal and basal OCR, was also significantly greater in MPN platelets (Figure 5B). Notably, ex vivo stimulation of MPN platelets with TRAP6 showed greater OCR responses than HI platelets, indicating a larger reserve capacity in the setting of increased energy demand (Figure 5C). We further identified positive correlations of basal respiration with P-selectin exposure and maximum respiration, suggesting the importance of mitochondrial respiration and energy supply in platelet hyperactivation (Figure 5D). Altogether, our results demonstrate elevated mitochondrial respiration and coupling of ATP generation in MPN platelets.

α-KG is a key intermediate of the TCA cycle, which is produced from isocitrate by oxidative decarboxylation or from glutamate by oxidative deamination (28). Recent studies found that α-KG supplementation alleviates aging and inflammation (29, 30). A recent report also showed that α-KG inhibits thrombosis and inflammation via suppression of AKT phosphorylation (31). Based on our findings of activation of OXPHOS and mTOR signaling along with decreased α-KG levels in MPN patient platelets, we tested whether α-KG supplementation could revert platelet metabolic alterations in the context of MPN. Indeed, α-KG incubation significantly reduced basal respiration, ATP generation, and reserve capacity in MPN platelets (Figure 5, E–G). α-KG inhibited the proton pump in ATP synthase as expected, reflected by increased proton leak as well as increased extracellular acidification rate (ECAR) (Figure 5, E–G). α-KG incubation also significantly reduced intracellular ATP levels in MPN platelets, consistent with our Seahorse results (Supplemental Figure 7C). Thus, α-KG supplementation modified the metabolic phenotype of platelets from MPN patients by inhibiting mitochondrial activity. A similar inhibition of mitochondrial respiration by α-KG was observed in UKE-1, a *JAK2*-mutant ET transformed acute leukemia cell line (Supplemental Figure 7, D and E).

### α-KG suppresses PI3K/AKT/mTOR signaling and platelet activation through ATP synthase inhibition.

**Figure 6:**
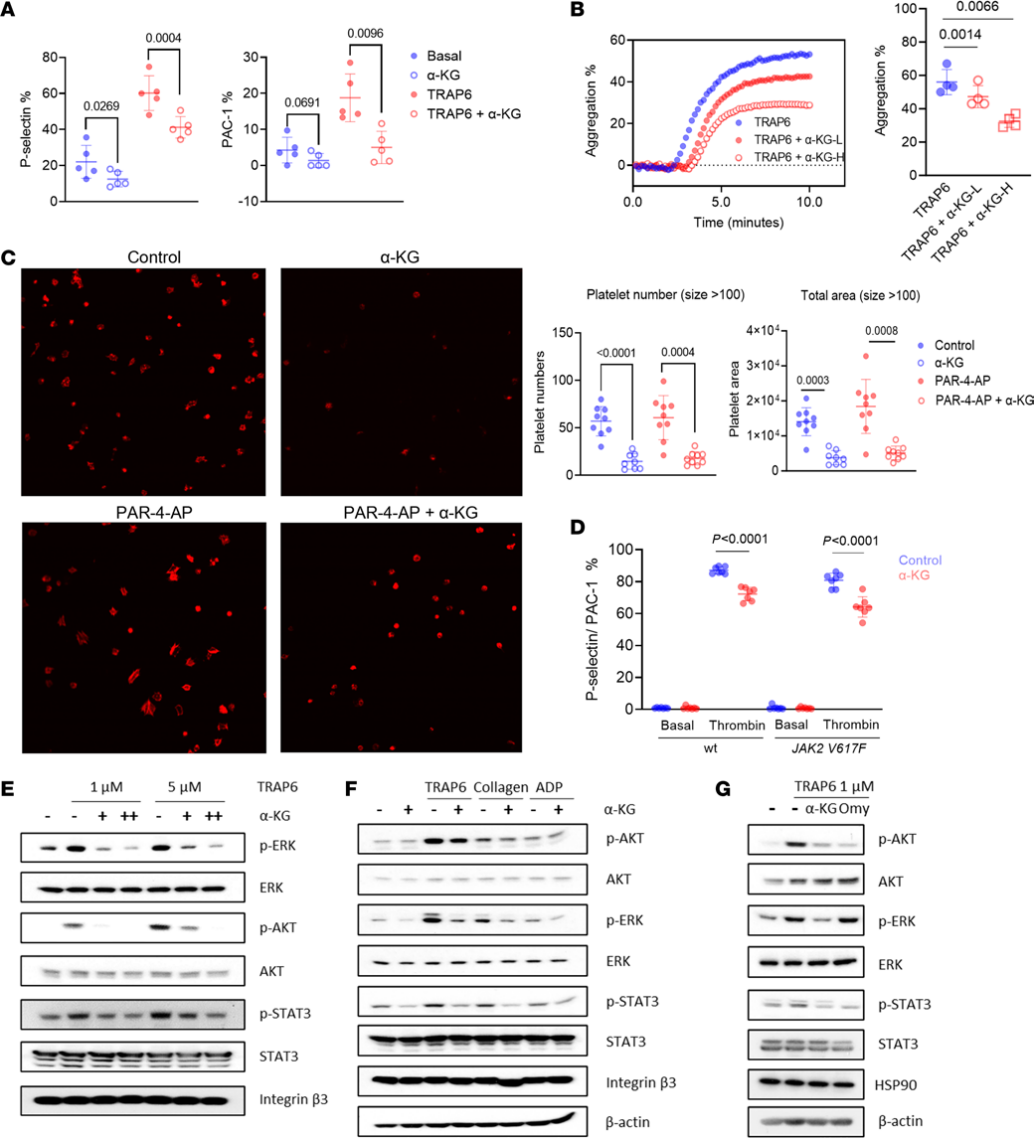
α-KG inhibited platelet activation through the suppression of ATP synthase. (**A**) Washed MPN platelet P-selectin and αIIbβ3 integrin expression changes after incubation with 200 μM octyl-α-KG for 1 hour ex vivo. Data are mean ± SD and were assessed by 2-tailed, paired Student’s *t* test. (**B**) Platelet aggregation assay with α-KG treatment. Washed ET platelets were incubated with 100 μM (L) or 200 μM (H) octyl-α-KG for 1 hour and stimulated with 5 μM TRAP6 followed by platelet aggregation tests. Maximal aggregation intensity was quantified as mean ± SD. Data were assessed by 2-tailed, paired Student’s *t* test. (**C**) Platelet adhesion and spreading assay with α-KG. Number and area of attached platelets on the coverslips were quantified with CellProfiler software (*n* = 9). Data are mean ± SD and were assessed by 2-tailed Mann-Whitney *U* test. Total original magnification, ×1000. (**D**) Platelet P-selectin and αIIbβ3 integrin expression changes in α-KG–supplemented mice. Age- and sex-matched wild-type and *Jak2 V617F*–knockin mice were supplemented with regular water (*n* = 7) or 2% α-KG (*n* = 7) for 1 week. Platelets were stimulated with thrombin ex vivo or not followed by flow cytometry analysis. Data shown as the ratio (mean ± SD) of P-selectin/αIIbβ3 integrin double-positive platelets and were assessed by 2-tailed Student’s *t* test. (**E**) Immunoblots of washed platelets after α-KG treatment. Washed ET platelets were incubated with 250 μM or 500 μM octyl-α-KG for 1 hour followed by stimulation with TRAP6 peptides. (**F**) Immunoblots of washed platelets after α-KG treatment with different stimulants. Washed ET platelets were incubated with 250 μM octyl-α-KG followed by stimulation with 5 μM TRAP6 peptides or 5 μg/mL collagen or 20 μM ADP. (**G**) Immunoblots of washed platelets after α-KG or oligomycin (Omy) treatment. Washed ET platelets were incubated with 250 μM octyl-α-KG or 1 μM Omy followed by stimulation with TRAP6 peptides.

After demonstrating the capacity of α-KG to inhibit platelet metabolism, we further evaluated the influence of α-KG on platelet activation. Ex vivo incubation of α-KG significantly reduced P-selectin expression and αIIbβ3 integrin activation following TRAP6 treatment of washed platelets from MPN patients (Figure 6A). A similar inhibitory effect of α-KG was observed in platelets from *Jak2 V617F* mice (Supplemental Figure 7F). Consistently, α-KG induced a dose-dependent inhibition of platelet aggregation (Figure 6B). Since αIIbβ3 downstream signaling (outside-in signaling) plays a critical role in platelet spreading and aggregation, we performed static platelet spreading and adhesion assays on platelets from *Jak2 V617F* mice. Ex vivo incubation with α-KG significantly decreased the number and area of platelets adhering to fibrinogen-coated surfaces; α-KG also inhibited platelet spreading induced by a PAR-4 agonist peptide (Figure 6C).

Supplementation with 2% α-KG in the drinking water inhibited P-selectin exposure and αIIbβ3 integrin activation on platelets in both wild-type and *Jak2 V617F*–knockin mice following thrombin stimulation (Figure 6D). To further investigate the inhibition of platelet activity by α-KG in vivo, we performed ferric chloride–induced (FeCl_3_-induced) vascular injury, a widely used model of thrombosis (32). α-KG supplementation (2%) led to a trend of delayed time to occlusion of the carotid artery in *Jak2 V617F*–knockin mice (Supplemental Figure 7, G and H). In addition, we observed significantly reduced tail thrombosis in 2% α-KG–supplemented BALB/c mice (Supplemental Figure 7, I and J), consistent with previous reports (31). Thus, our data demonstrated that α-KG inhibits MPN platelet hyperactivation and potentially lowers the risk of thrombosis.

Mechanistically, previous reports have shown that phosphorylation of STAT3, AKT, and ERK plays essential roles in platelet activation (33–35). Here, we found that α-KG inhibited p-STAT3, p-AKT, and p-ERK1/2 in MPN platelets in a dose-dependent manner following TRAP6 stimulation, without affecting the total amount of these proteins (Figure 6E). Moreover, α-KG downregulated p-STAT3, p-AKT, and p-ERK1/2 when platelets were activated with other agonists, including collagen and ADP (Figure 6F). Oligomycin, a widely used inhibitor of ATP synthase, showed similar downregulation of p-AKT and p-STAT3, suggesting a role for ATP synthase in mediating platelet activation (Figure 6G) (36). Similarly, addition of α-KG inhibited the proton pump of complex V in conjunction with decreased intracellular ATP levels, but increased mitochondrial membrane potential in the megakaryocytic MEG-01 cells as well as *JAK2*-mutant UKE-1 cells (Supplemental Figure 7, K and L). Evaluation of a potential effect of α-KG on apoptosis showed only a slight increase in phosphatidylserine exposure at a high dose of α-KG and no induction of cleaved caspase-3 (Supplemental Figure 6, C and D). α-KG slightly increased ROS levels in MPN platelets (Supplemental Figure 6G), consistent with previous reports in yeasts, fruit flies, and mice (37–39). To exclude possible nonspecific toxic effects of α-KG in cell lines, we also determined IC_50_ values for α-KG in MEG-01 (967.5 μM) and UKE-1 (785.6 μM) cell lines in cell viability assays (Supplemental Figure 7, M and N). Collectively, our data demonstrate that α-KG supplementation inhibits MPN platelet hyperactivation, in part due to inhibition of ATP synthase.

### α-KG exerts therapeutic effects on MPN and inhibits megakaryopoiesis.

**Figure 7:**
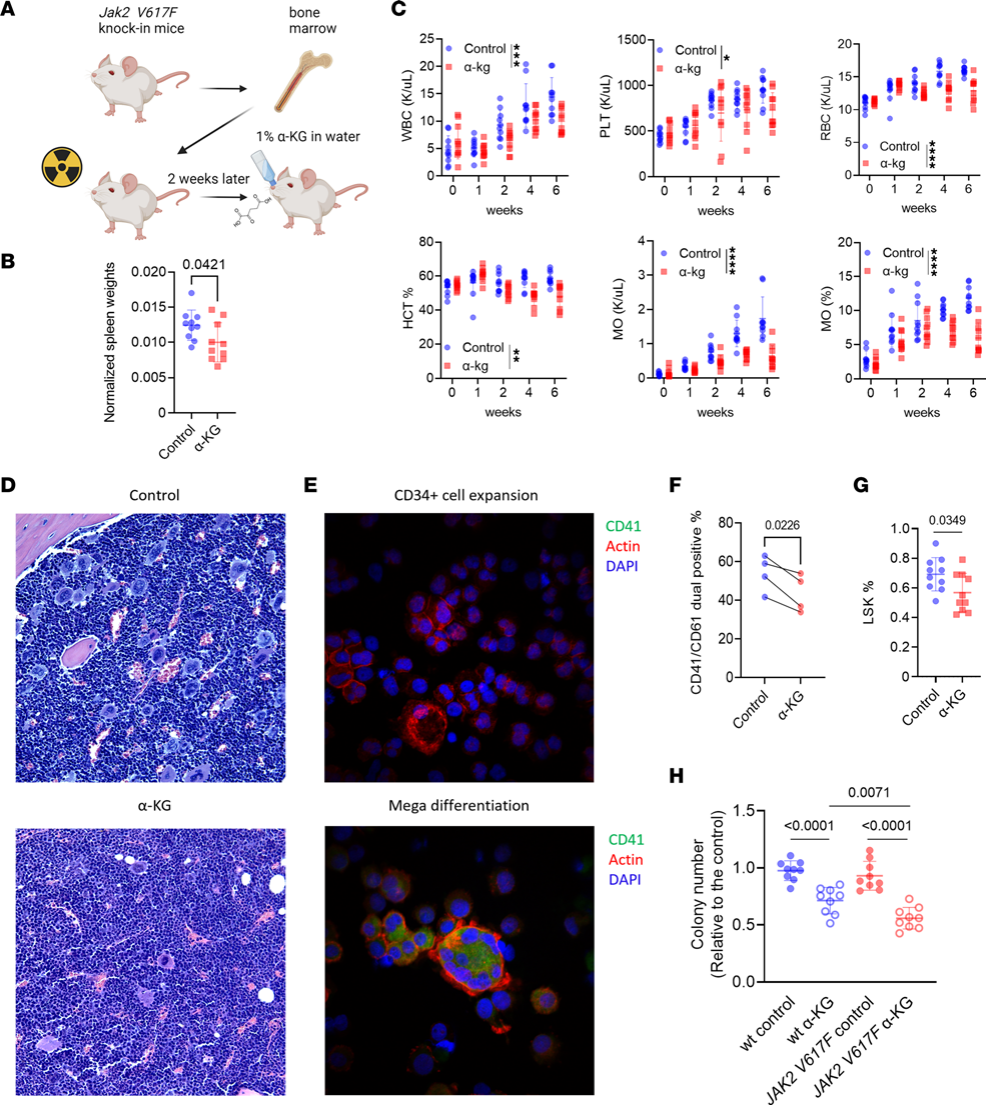
α-KG exerts therapeutic effects on MPN and inhibits megakaryopoiesis. (**A**) Schematic of the *Jak2 V617F*–knockin mice. cKit^+^ cells from *Jak2 V617F* CD45.2 C57BL/6J mice were isolated and transplanted into irradiated CD45.1 C57BL/6J mice. Two weeks after transplantation, mice were randomly grouped and supplemented with regular water (control, *n* = 10) or 1% α-KG in drinking water (*n* = 10) daily for 6 weeks. (**B**) Spleen weight of transplanted mice normalized to body weight measured at the end of treatments. Data are mean ± SD and were assessed by 2-tailed Student’s *t* test. (**C**) WBC, platelet (PLT) count, RBC, hematocrit (HCT), monocyte (MO) count, and ratio of *Jak2 V617F*–transplanted mice treated with regular water or α-KG across multiple time points. Data are mean ± SD and were assessed by 2-way ANOVA with Dunnett’s multiple-comparisons test. *P < 0.05, **P < 0.01, ***P < 0.001, ****P < 0.0001. (**D**) Representative images of H&E staining of femur bones from mice treated with regular water or α-KG. (**E**) Representative images of immunofluorescent staining of expanded CD34^+^ cells and differentiated megakaryocytes from the same individual. Total original magnification, ×200 (**D**) and ×600 (**E**). (**F**) Flow cytometry of CD41 and CD61 surface expression on in vitro megakaryocytes differentiated with α-KG. Sorted CD34^+^ hematopoietic stem and progenitor cells were cultured for megakaryocyte differentiation with 250 μM octyl-α-KG or DMSO control. CD41^+^CD61^+^ double-positive cells were determined by flow cytometry. Data are mean ± SD and were assessed by 2-tailed, paired Student’s *t* test. (**G**) Percentage of LSK cells from *Jak2 V617F*–transplanted mice treated with regular water or α-KG at the end of treatments. Data are mean ± SD and were assessed by 2-tailed Student’s *t* test. (**H**) CFU assays of mouse cKit^+^ cells with DMSO control or α-KG. Colony numbers were counted after 14 days. Data are mean ± SD and were assessed by 2-tailed Student’s *t* test.

After demonstrating the effects of α-KG on reducing platelet hyperreactivity ex vivo, we next sought to investigate the therapeutic impact of α-KG in MPNs in vivo. Mice engrafted with c-Kit^+^ cells from *Jak2 V617F*–knockin mice were supplemented with 1% α-KG in the drinking water versus regular water for 6 weeks (Figure 7A) (40). As expected, mice transplanted with *Jak2 V617F* cells developed characteristic MPN features, including splenomegaly, increased white blood cell (WBC), red blood cell (RBC), and platelet counts (Figure 7, B and C). α-KG supplementation markedly reduced splenomegaly, platelet, RBC, and monocyte counts without affecting body weight, lymphocytes, or B and T cell ratios compared to the control group (Figure 7, B and C, and Supplemental Figure 8A). Analysis of hematopoietic stem/progenitor cell (HSPC) subsets revealed a trend of decreased multipotent progenitor 2 (MPP2) cells in bone marrow from α-KG–supplemented mice, consistent with suppression of megakaryopoiesis and erythropoiesis (Supplemental Figure 8B). Observed increases in the MPP3 compartment suggest a compensatory upregulation of myeloid differentiation (Supplemental Figure 8B). α-KG treatment did not affect early lymphocyte specification, as the MPP4 compartment was not changed (Supplemental Figure 8B). Bone marrow histopathological analysis confirmed decreased megakaryocytes in α-KG–supplemented mice (Figure 7D). Importantly, α-KG supplementation in wild-type mice did not affect WBC or myeloid differentiation (Supplemental Figure 8C).

To further characterize the effects of α-KG on megakaryopoiesis, sorted human CD34^+^ cells were differentiated into megakaryocytes ex vivo with the addition of cytokines (Figure 7E). A decrease in CD41^+^CD61^+^ double-positive megakaryocytes was observed in the presence of α-KG, consistent with inhibition of megakaryopoiesis in α-KG–supplemented mice (Figure 7F). Moreover, α-KG inhibited megakaryocyte maturation of MEG-01 cells in vitro (Supplemental Figure 8, D and E). Notably, α-KG treatment also reduced Lin^−^Sca-1^+^c-Kit^+^ (LSK) cells in *Jak2* *V617F*– transplanted mice, suggesting a role of α-KG in HSPC regulation (Figure 7G). Finally, α-KG led to a reduction in myeloid colony formation from both wild-type and *Jak2 V617F* c-Kit^+^ cells, but to a larger degree in *Jak2 V617F* cells (Figure 7H).

RNA-seq analysis of *Jak2 V617F*–transplanted mice revealed that bone marrow cells of α-KG–supplemented mice clustered separately from control mice, suggesting differential gene expression induced by α-KG supplementation (Supplemental Figure 9A). GSEA showed inhibition of OXPHOS, mTOR, and myeloid differentiation pathways in α-KG–supplemented mice (Supplemental Figure 9, B–E). Expression of *Gata1* and *Epor*, critical mediators of platelet and RBC differentiation, were decreased in α-KG–supplemented mice (Supplemental Figure 9F). Downregulation of *Cdk1* and *Cdkn3* in α-KG–supplemented mice suggested cell cycle arrest (Supplemental Figure 9F). Reduced *Col1a1* and *Col1a2* transcripts in α-KG–supplemented mouse bone marrow suggested an effect on early fibrosis development (Supplemental Figure 9F), which was corroborated by decreased collagen immunofluorescent staining (Supplemental Figure 9, G and H). These findings collectively suggest that α-KG suppresses MPN disease features and megakaryopoiesis in *Jak2 V617F* mice.

### α-KG inhibits monocyte activation and hyperinflammation.

**Figure 8:**
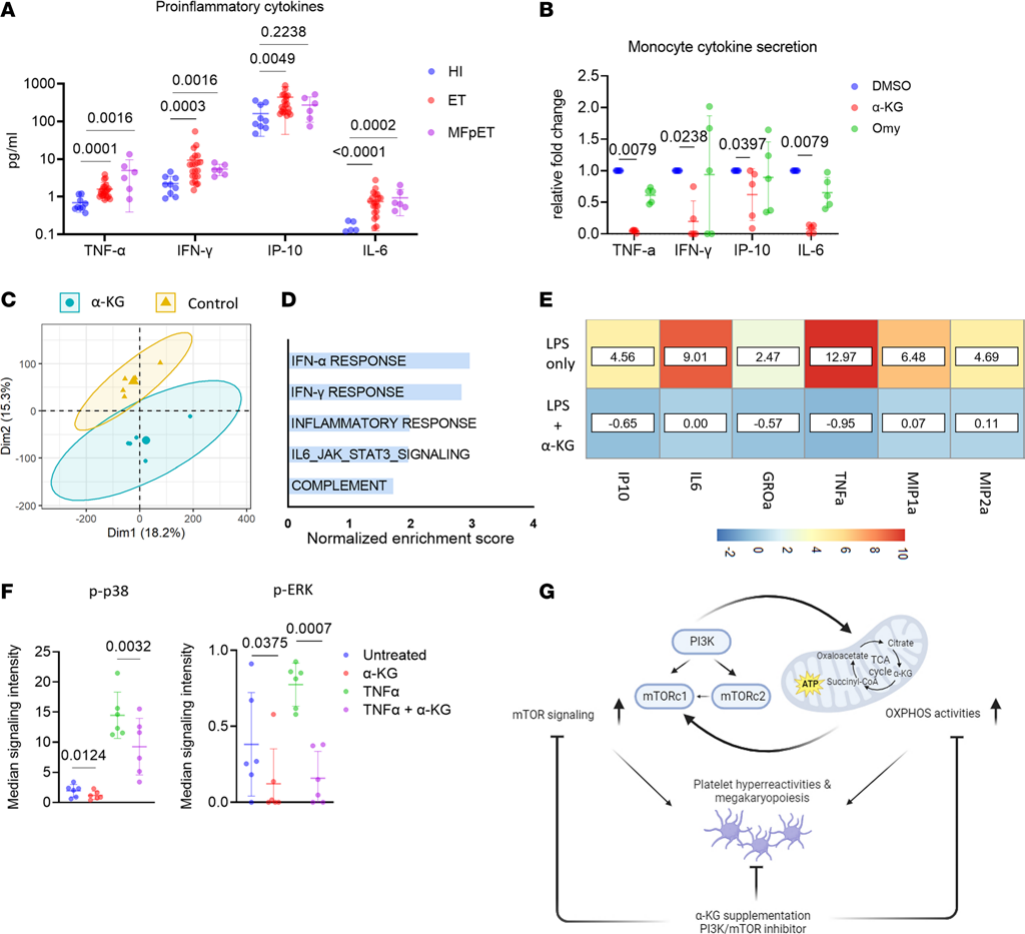
α-KG inhibited monocyte activation and hyperinflammation. (**A**) Plasma cytokine levels in HIs and MPN patients. Plasma from HIs (*n* = 9) and MPN patients (*n* = 28) was collected for the determination of 30 biomarkers using a V-PLEX Human Cytokine 30-Plex Kit from Meso Scale Discovery. Data are mean ± SD and were assessed by 2-tailed Mann-Whitney *U* test. MFpET, myelofibrosis post essential thrombocythemia. (**B**) Monocyte cytokine secretion changes with octyl-α-KG or oligomycin (Omy) treatment. Sorted CD14^+^ monocytes (0.5 × 10^6^) from MPN patients (*n* = 5) were incubated with octyl-α-KG or Omy for 8 hours and the supernatants were collected for cytokine determination by multiplex Luminex assay. Data are mean ± SD and were assessed by 2-tailed Mann-Whitney *U* test. (**C**) The PCA score plot of RNA-seq of sorted CD14^+^ monocytes after the incubation with octyl-α-KG or DMSO control. (**D**) Bar plot of GSEA results showing top 5 hallmark pathways enriched in DMSO- versus α-KG–treated monocytes. (**E**) Heatmap showing changes in cytokine secretion by CD11b^+^ myeloid cells from *Jak2 V617F* mice. Data were normalized to control group as fold changes. Enriched CD11b^+^ myeloid cells were incubated with LPS (0.1 mg/mL) in the presence or absence of α-KG for 6 hours. Supernatants were collected for cytokine determination by multiplex Luminex assay. (**F**) Dot plots of altered intracellular pathways of monocytes in peripheral blood of MPN patients by mass cytometry. Whole blood from MPN patients (*n* = 6) were incubated with octyl-α-KG or DMSO for 1 hour followed by stimulation with TNF-α. Data are mean ± SD and were assessed by 2-tailed, paired Student’s *t* test. (**G**) Proposed model showing the roles of PI3K/AKT/mTOR signaling and metabolic changes in platelets from MPN patients. A positive feedback loop involving PI3K/AKT/mTOR signaling and metabolic changes promotes platelet hyperreactivities and megakaryopoiesis in MPN. The supplementation of α-KG, which disrupts the feedback loop, shows therapeutic effects against platelet hyperreactivity, megakaryopoiesis, and chronic inflammation in MPN.

While MPN is initiated by the acquisition of mutations in HSPCs, its progression is driven, at least in part, by inflammation (10). We found significant elevation of proinflammatory cytokines in plasma from MPN patients, including TNF, IFN-γ, IP-10, and IL-6, compared with HIs (Figure 8A and Table 6). Previously, our group showed that monocytes play a critical role in hyperinflammation that characterizes MPNs (17, 18), many of which correlate with prognosis (41). As such, since monocyte counts decreased as early as 2 weeks in α-KG–supplemented mice (Figure 7C), we sought to investigate whether α-KG affected monocyte activation and inflammation.

Ex vivo incubation of α-KG with sorted CD14^+^ human monocytes significantly decreased the secretion of proinflammatory cytokines (Figure 8B). Bulk RNA-seq results demonstrated that α-KG downregulated genes involved in IFN pathways and inflammation response in monocytes (Figure 8, C and D). As shown in Supplemental Figure 10A, transcripts of inflammatory cytokines and chemokines, such as *CCL4*, *CCL8*, *CXCL11*, *CXCL10*, *IL1A*, *IL7*, and *IL15*, were downregulated in monocytes by α-KG. To address the effects of α-KG on inflammatory cytokine secretion in *Jak2 V617F* mice, enriched CD11b^+^ myeloid cells were incubated with LPS in the presence or absence of α-KG for 6 hours. Supernatants were collected for cytokine determination by multiplex Luminex assay. As shown in Figure 8E, α-KG inhibited the secretion of multiple proinflammatory cytokines by myeloid cells, including IP-10, IL-6, GROα, TNF-α, and MIPs. Mass cytometry analysis of whole blood from MPN patients revealed the inhibition of the MAPK signaling pathway in monocytes following α-KG treatment (Figure 8F and Supplemental Figure 10B). Similar effects of α-KG on the MAPK signaling pathway were found in U937, a monocytic cell line (Supplemental Figure 10C). Taken together, these findings suggest that α-KG inhibition of monocyte activation and inflammation might also contribute to its therapeutic effects in MPNs.

## Discussion

Thrombosis and bleeding complications are common in MPNs, but the underlying pathophysiology involved in these events is incompletely understood. Some reports have suggested platelet hyperreactivity and enhanced granule secretion as a potential mechanism for thrombosis in the setting of MPN (14, 19, 42–46). In this study, we used a multiomic profiling approach to characterize MPN platelets and uncovered functional, transcriptional, and metabolic signatures associated with platelet hyperreactivity. Importantly, we identified the PI3K/AKT/mTOR signaling pathway as a significant contributor to the platelet dysregulation observed in MPN patients. We further demonstrated that both direct inhibition of the PI3K/AKT/mTOR signaling pathway and metabolic intervention via α-KG supplementation suppress platelet activation. Importantly, our results suggest therapeutic potential of α-KG supplementation to prevent platelet hyperreactivity in MPN patients.

We chose ET samples for scRNA-seq based on several considerations. ET samples showed the highest PLA formation as well as P-selectin upregulation among MPNs in flow cytometry analysis. Higher platelet counts in ET samples provided better platelet sampling as well as increased confidence and reliability for platelet analysis. To confirm our scRNA-seq observations, we validated the results using publicly available data sets as shown above (26). Consistent with our results, a recent large-scale platelet RNA-seq profiling also suggested altered immune, metabolic, and proteostatic pathways in all 3 MPN subtypes, with the MPN platelet transcriptome robustly predicting disease progression, therefore highlighting the importance of platelet biology in MPNs (47). Although PI3K/AKT/mTOR signaling has been established as a key regulator of proliferation, cancer, longevity, and mitochondrial homeostasis, its role in mediating platelet hyperreactivity in MPNs has not been previously reported (48). Our multiomic and functional results identified PI3K/AKT/mTOR signaling as a key driver of platelet hyperreactivity in MPNs. The effects of dual PI3K/mTOR inhibition by omipalisib underscores the essential role of PI3K/AKT/mTOR signaling in mediating platelet metabolism and hyperactivation in MPNs, suggesting potential therapeutic role in MPNs (49, 50). We also identified metabolic changes consisting of enhanced OXPHOS and glycolysis activity in MPN platelets. We further demonstrate that α-KG supplementation is an effective metabolic intervention for MPNs, inhibiting both PI3K/AKT/mTOR signaling and mitochondrial activation. Thus, our work reveals PI3K/AKT/mTOR signaling and metabolic changes as the major drivers of platelet reactivity in MPNs.

Metabolic changes in MPNs have been previously reported. It has been shown that glutaminase inhibitors suppress the growth of *JAK2* *V617F*–mutant cell lines and MPN patient CD34^+^ cells (51). IDH2 inhibitors showed efficacy in cells from MPN patients carrying both *JAK2* and *IDH2* mutations (52). Recently, elevated glycolysis in *JAK2*-mutant HSPCs was identified as a novel target to treat MPNs (27). It is plausible that mature blood cells in MPNs, such as monocytes and platelets, may also exhibit aberrant metabolism and contribute to disease development and progression. Our multiomic results suggest enhanced OXPHOS activities in MPN platelets, validated by increased rates of basal respiration and ATP generation observed in bioenergetic analysis. In this work, we showed that mitochondrial abnormalities fuel platelet hyperactivation in MPNs, with both increased basal respiration and reserve capacity after TRAP6 stimulation. Of note, our results suggest that the PI3K/AKT/mTOR signaling serves as a driver of mitochondrial abnormalities in MPN platelets, as the inhibition of mitochondrial activities by α-KG supplementation decreased pathway activation.

α-KG has been shown to be highly versatile in regulating cellular activities, as evidenced by the extensive list of described α-KG–dependent proteins, including Jumonji domain–containing histone demethylases, TET proteins mediating DNA methylation, and prolyl-hydroxylase domain enzymes degrading hypoxia-inducible factor proteins (28). Previous reports have demonstrated that α-KG suppresses mitochondrial activities by directly inhibiting ATP synthase and further affecting mTOR signaling (29). Our results show that α-KG and oligomycin, a specific ATP synthase inhibitor, both inhibit phosphorylation of AKT and STAT3 in platelets. Our data also show that both α-KG and mTOR inhibitors inhibit platelet activation. Thus, it is very likely that suppression of mitochondrial activities contributes to inhibition of platelet signaling by α-KG. Another possible mechanism is that α-KG, as a cofactor of prolyl hydrolase 2 (PHD2), promotes PHD2 activity through an elevated intracellular α-KG–to-succinate ratio and further suppresses phosphorylation of AKT (31). Since platelets are anucleate, the histone/DNA methylation mechanism can be reasonably excluded. However, we cannot exclude other possible mechanisms, as discussed in a recent review (53). Further research is needed to explore targets and mechanisms of α-KG in human cells. α-KG has also been previously reported to maintain embryonic stem cell pluripotency via epigenetic regulation, but its effects on hematopoiesis have been unexplored (54). Although α-KG supplementation decreased collagen transcripts and staining in *Jak2 V617F*–transplanted mice, effects of α-KG supplementation on bone marrow fibrosis merit further investigation. In this study, we demonstrated inhibition of myeloid differentiation in *Jak2 V617F*–transplanted mice mice but not in wild-type mice, indicating the effectiveness and safety of α-KG supplementation in the MPN setting. We also observed inhibition of monocyte activation and cytokine secretion by α-KG treatment, in agreement with the previously described role of α-KG in alleviating inflammation and oxidative stress (55, 56). Therefore, the inhibition of myeloid differentiation, which resulted in reduced platelet, RBC, and monocyte counts, in α-KG–treated mice could also be a result of decreased chronic inflammation in MPNs.

In summary, our data reveal a previously unrecognized mitochondrial disorder in platelets from MPN patients; this energetic alteration leads to platelet hyperreactivity potentially contributing to thrombotic events (Figure 8G). We also identified aberrant PI3K/AKT/mTOR signaling partially rectified by α-KG supplementation (Figure 8G). These findings may lead to novel therapeutic approaches targeting platelet hyperreactivity and chronic inflammation in MPN.

## Methods

### Cell culture

MEG-01 (ATCC) and UKE-1 (Coriell Institute) cells were cultured in RPMI 1640 medium (ATCC modification) and RPMI 1640 medium (Thermo Fisher Scientific), respectively, supplemented with 10% fetal bovine serum (FBS) and 1% penicillin/streptomycin. All cell lines were maintained at 37°C and 5% CO_2_ and regularly tested for mycoplasma.

### Blood collection and platelet isolation from humans

Whole blood was drawn into 4.5 mL tubes containing buffered sodium citrate in accordance with an Institutional Review Board–approved protocol at Washington University in St. Louis. Blood was transferred into 15 mL tubes with the addition of 10% (v/v) prewarmed citrate-dextrose solution (ACD). Platelet-rich plasma (PRP) was separated from the other cellular components of blood by centrifugation for at 250*g* for 20 minutes without brake and then carefully withdrawn using a plastic Pasteur pipette without disturbing the buffy coat. Prostaglandin E1 (PGE1) was added to PRP to a final concentration of 1 μM to prevent aggregation and then spun again for 10 minutes at 1000*g* without brake. The platelet pellet was then carefully washed with 5 mL of Tyrode’s-ACD, which consisted of 9 parts Modified Tyrode’s buffer (129 mM NaCl, 0.34 mM Na_2_HPO_4_, 2.9 mM KCl, 12 mM NaHCO_3_, 20 mM HEPES, 5 mM glucose, 1 mM MgCl_2_; pH 7.3) and 1 part ACD in the presence of PGE1, and spun for 5 minutes at 700*g*. The resultant pellet was gently resuspended in Modified Tyrode’s buffer and adjusted to a count of 3 × 10^8^ platelets/mL for subsequent experiments.

### PLA and platelet activation analysis by flow cytometry

PLAs were tested as previously described (57). Whole blood samples were diluted 5-fold by adding HEPES buffer (145 mM NaCl, 5 mM KCl, 1 mM MgSO_4_, 0.5 mM NaH_2_PO_4_, 5 mM glucose, 10 mM HEPES/Na) within 30 minutes of collection. Diluted blood samples were incubated with PerCP-conjugated anti-CD61 (BioLegend) and Pacific Blue–conjugated anti-CD45 mAb (BioLegend) for PLA analysis. Samples were also stained with APC-conjugated anti-CD62P (AK4, BioLegend) and FITC-conjugated anti-CD41/CD61 (PAC-1, BioLegend). See Supplemental Table 2 for a list of all antibodies used in the study. Samples were stained for 10 minutes at room temperature. For platelet activation, 1 μM TRAP6 (4031274, Bachem) or 5 μg/mL collagen (P/N 385, Chrono Log) stimulation was added simultaneously with antibody staining. Next, samples were fixed with 1.5 % paraformaldehyde, followed by 4.6-fold dilution with distilled water for RBC lysis. Samples were analyzed by flow cytometry.

### Platelet aggregation assay

Light transmission aggregometry of washed human platelets (300 × 10^3^/μL) was performed in a PAP-8E platelet aggregometer (Biodata Corporation). Aggregation was induced with TRAP6 or collagen as indicated in each experiment.

### scRNA-seq and analysis

All MPN patients in the cohort were recruited at the Hematology Department of Washington University School of Medicine in St. Louis. Sample processing was performed as previously published (58, 59). 10× Genomics Chromium Next GEM Single cell 3′ Reagent v3.1 (Dual Index) was used for GEM Generation, barcoding, and cDNA library preparation per manufacturer’s guidelines, and cDNA was sequenced using the 10× Genomics Single-Cell RNA-Seq platform at the Genome Technology Access Center at the McDonnell Genome Institute of Washington University in St. Louis. scRNA-seq analysis details are provided in Supplemental Methods.

### Sample preparation for metabolomics and proteomics analyses

Washed platelets were isolated as described above without adding glucose or other nutrients in Modified Tyrode’s buffer. Platelets (3 × 10^8^) were carefully washed with PBS twice without flushing or pipetting the pellet to remove all extraneous metabolites. Pellets were flash frozen with liquid nitrogen and stored at –80°C. Metabolomics and proteomics were performed as previously published and details are provided in Supplemental Methods (60).

### Seahorse assay

Bioenergetics of washed platelets (20 × 10^6^/well) were determined by Seahorse XFe96 (Agilent Technologies), as previously described (61). After measurement of basal OCR, OCR due to proton leak was determined by oligomycin A (2.5 μM) treatment. Maximal uncoupled OCR was measured by the addition of the uncoupler carbonyl cyanide *p*-(trifluoromethoxy) phenylhydrazone (FCCP; 0.5 μM). Nonmitochondrial OCR (defined as the OCR of all cellular processes excluding mitochondrial respiration) was measured in the presence of rotenone/antimycin A (1 μM). In subsets of samples, TRAP6 (20 μM) was added before oligomycin A to measure TRAP6-stimulated energy demand. In subsets of samples, platelets were preincubated with octyl-α-KG or other reagents as indicated for 1 hour to determine their effects on platelet bioenergetics.

### Western blotting

Washed platelets were lysed with RIPA buffer (Thermo Fisher Scientific) with protease and phosphatase inhibitor cocktail (Thermo Fisher Scientific) and quantified using the Bradford assay (Thermo Fisher Scientific). Boiled protein (20 μg) was loaded for detection as previously described (62). Quantification of Western blot bands was performed using ImageJ software (NIH).

### Platelet adhesion and spreading assay

Platelets were isolated from *Jak2* *V617F*–knockin mice by sequential centrifugation. Platelets were incubated with 250 μM octyl-α-KG at 37°C or DMSO control for 1 hour followed by 100 μM PAR-4 agonist peptide for 5 minutes. Treated platelets were incubated on fibrinogen-coated coverslips for 45 minutes at 37°C in wells of a 24-well plate. Coverslips were washed 3 times with PBS, fixed with paraformaldehyde, permeabilized with 0.1% Triton X-100, and mounted with VECTASHIELD antifade mounting medium with phalloidin (Vector Laboratories) and imaged using a Nikon A1Rsi confocal microscope and NIS-Elements AR software. Number and area of attached platelets on the coverslips were quantified with CellProfiler software (*n* = 9).

### Colony-forming unit assays

Colony-forming unit (CFU) assays were performed in semisolid culture using Methocult M3434 (STEMCELL Technologies) containing IL-3, IL-6, stem cell factor, and erythropoietin. Sorted cKit^+^ cells were seeded at 1000 cells/mL with 250 μM octyl-α-KG or DMSO control in triplicate. Colonies were counted 14 days after seeding.

### Megakaryocyte differentiation and proplatelet formation

CD34^+^ cells were sorted from MPN patient and HI cryopreserved bone marrow mononuclear cells (BMMCs) with MicroBeads (Miltenyi Biotec). Sorted cells (2 × 10^4^) were plated in triplicate in serum-free media containing StemSpan Megakaryocyte Expansion Supplement (STEMCELL Technologies) for 10–12 days. Media were replaced every 5 days. Megakaryocyte cell surface markers CD41 (FITC-conjugated anti-CD41; X, X) and CD61 (PerCP-conjugated anti-CD61; X, X) were measured by flow cytometry. MEG-01 cells were treated with 10 ng/mL phorbol 12-myristate 13-acetate (PMA) to induce megakaryocyte differentiation (63).

### Plasma cytokine analysis

Peripheral blood plasma collected under standard protocols was stored at −80°C. Concentrations of 30 cytokines/chemokines were analyzed in duplicate using the Meso Scale Discovery platform with the V-PLEX Human Cytokine 30-Plex Kit (Meso Scale Discovery). Statistical analysis was performed using Prism (GraphPad Software).

### In vivo models

#### Wild-type mice.

Regular water or 1% dietary α-KG was administered to 7 week-old C57BL/6J mice (stock 000664, The Jackson Laboratory). Mice were treated for 4 weeks and peripheral blood was collected every week for Hemavet analysis (Drew Scientific).

#### Jak2 V617F model.

cKit^+^ cells from CD45.2 *Jak2* *V617F* donor mice were transplanted into lethally irradiated recipient CD45.1 mice as previously described (40, 59, 64). Two weeks after transplantation, mice were supplemented with regular water or 1% dietary α-KG for 6 weeks. Peripheral blood was collected every other week for Hemavet tests. Mice were sacrificed at endpoint, and body, spleen, and liver weights were recorded. Bone marrow samples were collected for flow cytometry and bulk RNA-seq analysis. Femur bones were collected for hematoxylin and eosin (H&E) staining.

For platelet activation experiments, *Jak2* *V617F* mice were supplemented with regular water (*n* = 7) or 2% α-KG (*n* = 7) for 1 week. Peripheral blood was collected for platelet activation analysis as described above.

### Statistics

Statistical analyses were performed using GraphPad Prism and R software (https://www.r-project.org/). Two-tailed Student’s *t* test, Mann-Whitney *U* test, 1-way ANOVA, 2-way ANOVA, and Pearson’s correlations were performed as indicated. All relevant assays were performed independently at least 3 times. A *P* value of 0.05 or less was considered significant.

### Study approval

Patient and HI control peripheral blood or bone marrow samples were obtained with written consent according to a protocol approved by the Washington University Human Studies Committee (WU no. 01-1014) and to the Helsinki Declaration of the World Medical Association. Mononuclear cells (PBMCs or BMMCs) were obtained by Ficoll gradient extraction and cryopreserved according to standard procedures. Additional BMMCs were purchased from STEMCELL Technologies. Lists of patient samples utilized in this study are provided in Tables 1 and 3–6. All mouse procedures were conducted in accordance with the Institutional Animal Care and Use Committee of Washington University (no. 20-0463).

### Data availability

We have submitted the scRNA-seq and bulk RNA-seq data sets to the NCBI Gene Expression Omnibus (GEO GSE244590). More detailed information for this paper can be found in the supplemental materials. Additional data are available in the supplemental Supporting Data Values file and from the corresponding author upon reasonable request.

## Author contributions

FH, ABAL, TK, AL, SL, and LY performed experiments. KJA, NML, DACF, LAH, MB, BG, and MCF provided technical support. MJC coordinated clinical sample collection. ADA performed proteomics and metabolomics experiments. FH, SMS, JDP, and STO designed and supervised the experiments. FH, JDP, and STO wrote the manuscript. All authors read and approved of the manuscript.

## Supplementary Material

Supplemental data

Supplemental data set 1

Supplemental data set 2

Supplemental table 2

Supporting data values

## Figures and Tables

**Table 6:**
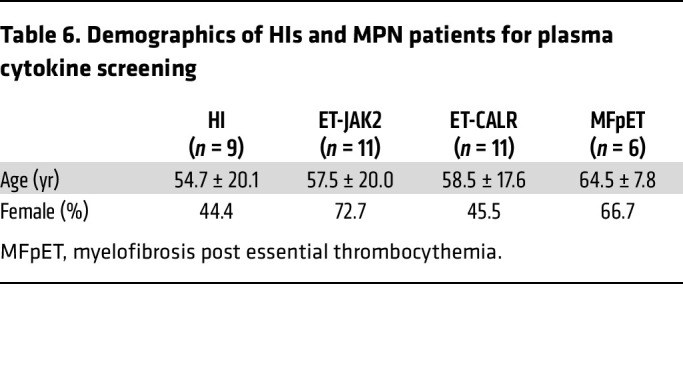
Demographics of HIs and MPN patients for plasma cytokine screening

**Table 1:**
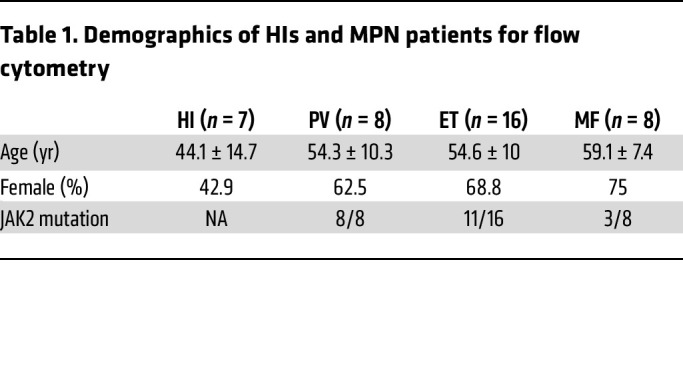
Demographics of HIs and MPN patients for flow cytometry

**Table 2:**
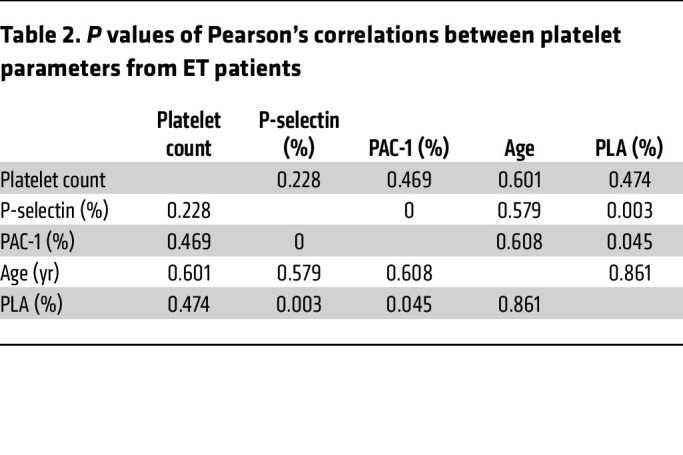
*P* values of Pearson’s correlations between platelet parameters from ET patients

**Table 3:**
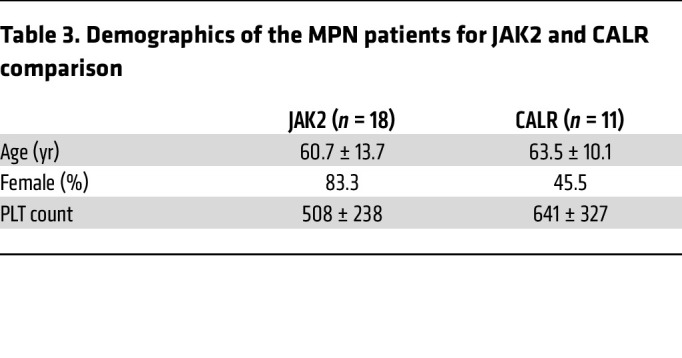
Demographics of the MPN patients for JAK2 and CALR comparison

**Table 4:**
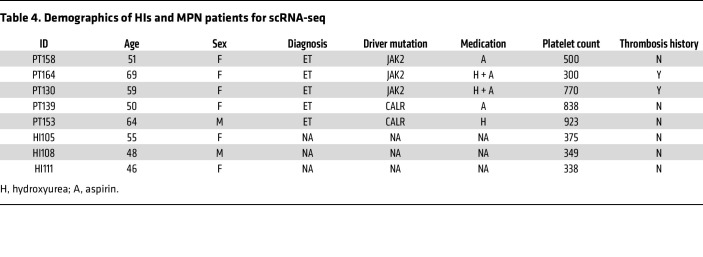
Demographics of HIs and MPN patients for scRNA-seq

**Table 5:**
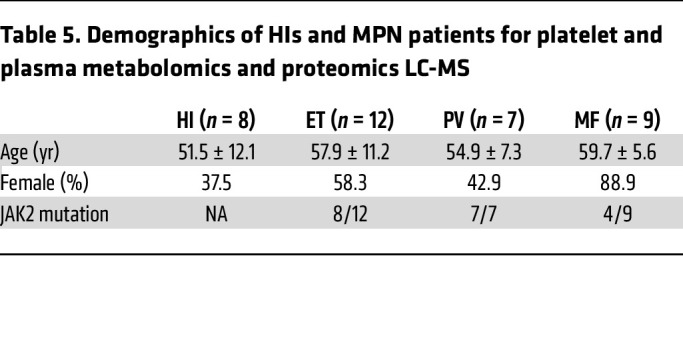
Demographics of HIs and MPN patients for platelet and plasma metabolomics and proteomics LC-MS

## Version 1 12/07/2023

In-Press Preview

## Version 2 02/01/2024

Electronic publication

## References

[1] Barbui T (2018). The 2016 WHO classification and diagnostic criteria for myeloproliferative neoplasms: document summary and in-depth discussion. Blood Cancer J.

[2] Rungjirajittranon T (2019). A systematic review and meta-analysis of the prevalence of thrombosis and bleeding at diagnosis of Philadelphia-negative myeloproliferative neoplasms. BMC Cancer.

[3] Barbui T (2022). Thrombosis in myeloproliferative neoplasms during cytoreductive and antithrombotic drug treatment. Res Pract Thromb Haemost.

[4] Hasselbalch HC (2021). The pathobiology of thrombosis, microvascular disease, and hemorrhage in the myeloproliferative neoplasms. Blood.

[5] Duchemin J (2010). Increased circulating procoagulant activity and thrombin generation in patients with myeloproliferative neoplasms. Thromb Res.

[6] Panova-Noeva M (2011). Platelet-induced thrombin generation by the calibrated automated thrombogram assay is increased in patients with essential thrombocythemia and polycythemia vera. Am J Hematol.

[7] Cella G (2010). Nitric oxide derivatives and soluble plasma selectins in patients with myeloproliferative neoplasms. Thromb Haemost.

[8] Hobbs CM (2013). JAK2V617F leads to intrinsic changes in platelet formation and reactivity in a knock-in mouse model of essential thrombocythemia. Blood.

[9] Lamrani L (2014). Hemostatic disorders in a JAK2V617F-driven mouse model of myeloproliferative neoplasm. Blood.

[10] Fisher DAC (2021). Inflammatory pathophysiology as a contributor to myeloproliferative neoplasms. Front Immunol.

[11] Taus F (2020). Platelets promote thromboinflammation in SARS-CoV-2 pneumonia. Arterioscler Thromb Vasc Biol.

[12] Sefer D (2022). Correlation between leukocyte-platelet aggregates and thrombosis in myeloproliferative neoplasms. Int J Lab Hematol.

[13] Marin Oyarzun CP, Heller PG (2019). Platelets as mediators of thromboinflammation in chronic myeloproliferative neoplasms. Front Immunol.

[14] Marin Oyarzun CP (2020). Platelet Toll-like receptors mediate thromboinflammatory responses in patients with essential thrombocythemia. Front Immunol.

[15] Wang L (2017). Platelet mitochondrial dysfunction and the correlation with human diseases. Biochem Soc Trans.

[16] Davizon-Castillo P (2019). TNF-α-driven inflammation and mitochondrial dysfunction define the platelet hyperreactivity of aging. Blood.

[17] Fisher DAC (2017). Mass cytometry analysis reveals hyperactive NF kappa B signaling in myelofibrosis and secondary acute myeloid leukemia. Leukemia.

[18] Fisher DAC (2019). Cytokine production in myelofibrosis exhibits differential responsiveness to JAK-STAT, MAP kinase, and NFκB signaling. Leukemia.

[19] Jensen MK (2001). Increased circulating platelet-leukocyte aggregates in myeloproliferative disorders is correlated to previous thrombosis, platelet activation and platelet count. Eur J Haematol.

[20] Rotunno G (2014). Impact of calreticulin mutations on clinical and hematological phenotype and outcome in essential thrombocythemia. Blood.

[21] Rumi E (2014). JAK2 or CALR mutation status defines subtypes of essential thrombocythemia with substantially different clinical course and outcomes. Blood.

[22] Garcia-Alonso L (2019). Benchmark and integration of resources for the estimation of human transcription factor activities. Genome Res.

[23] Lee WY (2017). GATA1 is a sensitive and specific nuclear marker for erythroid and megakaryocytic lineages. Am J Clin Pathol.

[24] Frith K (2021). The role of ZEB2 in human CD8 T lymphocytes: clinical and cellular immune profiling in Mowat-Wilson syndrome. Int J Mol Sci.

[25] Huang Z (2007). STAT1 promotes megakaryopoiesis downstream of GATA-1 in mice. J Clin Invest.

[26] Gnatenko DV (2005). Platelets express steroidogenic 17beta-hydroxysteroid dehydrogenases. Distinct profiles predict the essential thrombocythemic phenotype. Thromb Haemost.

[27] Rao TN (2019). JAK2-mutant hematopoietic cells display metabolic alterations that can be targeted to treat myeloproliferative neoplasms. Blood.

[28] Wu N (2016). Alpha-ketoglutarate: physiological functions and applications. Biomol Ther (Seoul).

[29] Chin RM (2014). The metabolite α-ketoglutarate extends lifespan by inhibiting ATP synthase and TOR. Nature.

[30] Asadi Shahmirzadi A (2020). Alpha-ketoglutarate, an endogenous metabolite, extends lifespan and compresses morbidity in aging mice. Cell Metab.

[31] Shrimali NM (2021). α-ketoglutarate inhibits thrombosis and inflammation by prolyl hydroxylase-2 mediated inactivation of phospho-Akt. EBioMedicine.

[32] Jones WL (2022). Apolipoprotein A-I, elevated in trauma patients, inhibits platelet activation and decreases clot strength. Platelets.

[33] Zhou Z (2013). Signal transducer and activator of transcription 3 (STAT3) regulates collagen-induced platelet aggregation independently of its transcription factor activity. Circulation.

[34] Chen J (2004). Impaired platelet responses to thrombin and collagen in AKT-1-deficient mice. Blood.

[35] Flevaris P (2009). Two distinct roles of mitogen-activated protein kinases in platelets and a novel Rac1-MAPK-dependent integrin outside-in retractile signaling pathway. Blood.

[36] Hao W (2010). Oligomycin-induced bioenergetic adaptation in cancer cells with heterogeneous bioenergetic organization. J Biol Chem.

[37] Bayliak M (2017). Growth on alpha-ketoglutarate increases oxidative stress resistance in the yeast Saccharomyces cerevisiae. Int J Microbiol.

[38] Su Y (2019). Alpha-ketoglutarate extends Drosophila lifespan by inhibiting mTOR and activating AMPK. Aging (Albany NY).

[39] Niemiec T (2011). Alpha-ketoglutarate stabilizes redox homeostasis and improves arterial elasticity in aged mice. J Physiol Pharmacol.

[40] Mullally A (2010). Physiological Jak2V617F expression causes a lethal myeloproliferative neoplasm with differential effects on hematopoietic stem and progenitor cells. Cancer Cell.

[41] Masselli E (2020). Cytokine profiling in myeloproliferative neoplasms: overview on phenotype correlation, outcome prediction, and role of genetic variants. Cells.

[42] Arellano-Rodrigo E (2006). Increased platelet and leukocyte activation as contributing mechanisms for thrombosis in essential thrombocythemia and correlation with the JAK2 mutational status. Haematologica.

[43] Falanga A (2005). Leukocyte-platelet interaction in patients with essential thrombocythemia and polycythemia vera. Exp Hematol.

[44] Robertson B (2007). Platelet and coagulation activation markers in myeloproliferative diseases: relationships with JAK2 V6I7 F status, clonality, and antiphospholipid antibodies. J Thromb Haemost.

[45] Trappenburg MC (2009). Elevated procoagulant microparticles expressing endothelial and platelet markers in essential thrombocythemia. Haematologica.

[46] Alvarez-Larran A (2008). Increased platelet, leukocyte, and coagulation activation in primary myelofibrosis. Ann Hematol.

[47] Shen Z (2021). Platelet transcriptome identifies progressive markers and potential therapeutic targets in chronic myeloproliferative neoplasms. Cell Rep Med.

[48] Gao M (2011). Phosphatidylinositol 3-kinase affects mitochondrial function in part through inducing peroxisome proliferator-activated receptor γ coactivator-1β expression. Br J Pharmacol.

[49] Khan I (2013). AKT is a therapeutic target in myeloproliferative neoplasms. Leukemia.

[50] Moyo TK (2023). PI3K inhibition restores and amplifies response to ruxolitinib in patients with myelofibrosis. Clin Cancer Res.

[51] Zhan H (2015). Targeting glutamine metabolism in myeloproliferative neoplasms. Blood Cells Mol Dis.

[52] McKenney AS (2018). JAK2/IDH-mutant-driven myeloproliferative neoplasm is sensitive to combined targeted inhibition. J Clin Invest.

[53] Naeini SH (2023). Alpha-ketoglutarate as a potent regulator for lifespan and healthspan: evidences and perspectives. Exp Gerontol.

[54] Carey BW (2015). Intracellular α-ketoglutarate maintains the pluripotency of embryonic stem cells. Nature.

[55] Mailloux RJ (2009). Alpha-ketoglutarate dehydrogenase and glutamate dehydrogenase work in tandem to modulate the antioxidant alpha-ketoglutarate during oxidative stress in Pseudomonas fluorescens. J Bacteriol.

[56] Ali R (2012). Ameliorative potential of alpha-ketoglutaric acid (AKG) on acute lung injuries induced by ammonia inhalation in rats. Exp Lung Res.

[57] Nagasawa A (2013). The basis examination of leukocyte-platelet aggregates with CD45 gating as a novel platelet activation marker. Int J Lab Hematol.

[58] Fisher MH (2020). ETV6 germline mutations cause HDAC3/NCOR2 mislocalization and upregulation of interferon response genes. JCI Insight.

[59] Kong T (2023). DUSP6 mediates resistance to JAK2 inhibition and drives leukemic progression. Nat Cancer.

[60] D’Alessandro A (2020). Metabolic phenotypes of standard and cold-stored platelets. Transfusion.

[61] Nguyen QL (2017). Platelets from pulmonary hypertension patients show increased mitochondrial reserve capacity. JCI Insight.

[62] He F (2019). Interaction between p53 N terminus and core domain regulates specific and nonspecific DNA binding. Proc Natl Acad Sci U S A.

[63] Schlinker AC (2016). Megakaryocyte polyploidization and proplatelet formation in low-attachment conditions. Biochem Eng J.

[64] Kong T (2022). Pevonedistat targets malignant cells in myeloproliferative neoplasms in vitro and in vivo via NFκB pathway inhibition. Blood Adv.

