# Supplementary material for: Multiomic profiling reveals metabolic alterations mediating aberrant platelet activity and inflammation in myeloproliferative neoplasms
Source: J Clin Invest. 2024 Feb 1;134(3):e172256. doi: 10.1172/JCI172256 (PMC10836808; doi:10.1172/JCI172256)
Supplement: Supplemental data [file jci-134-172256-s015.pdf]

Supplementary Figures

Figure S1

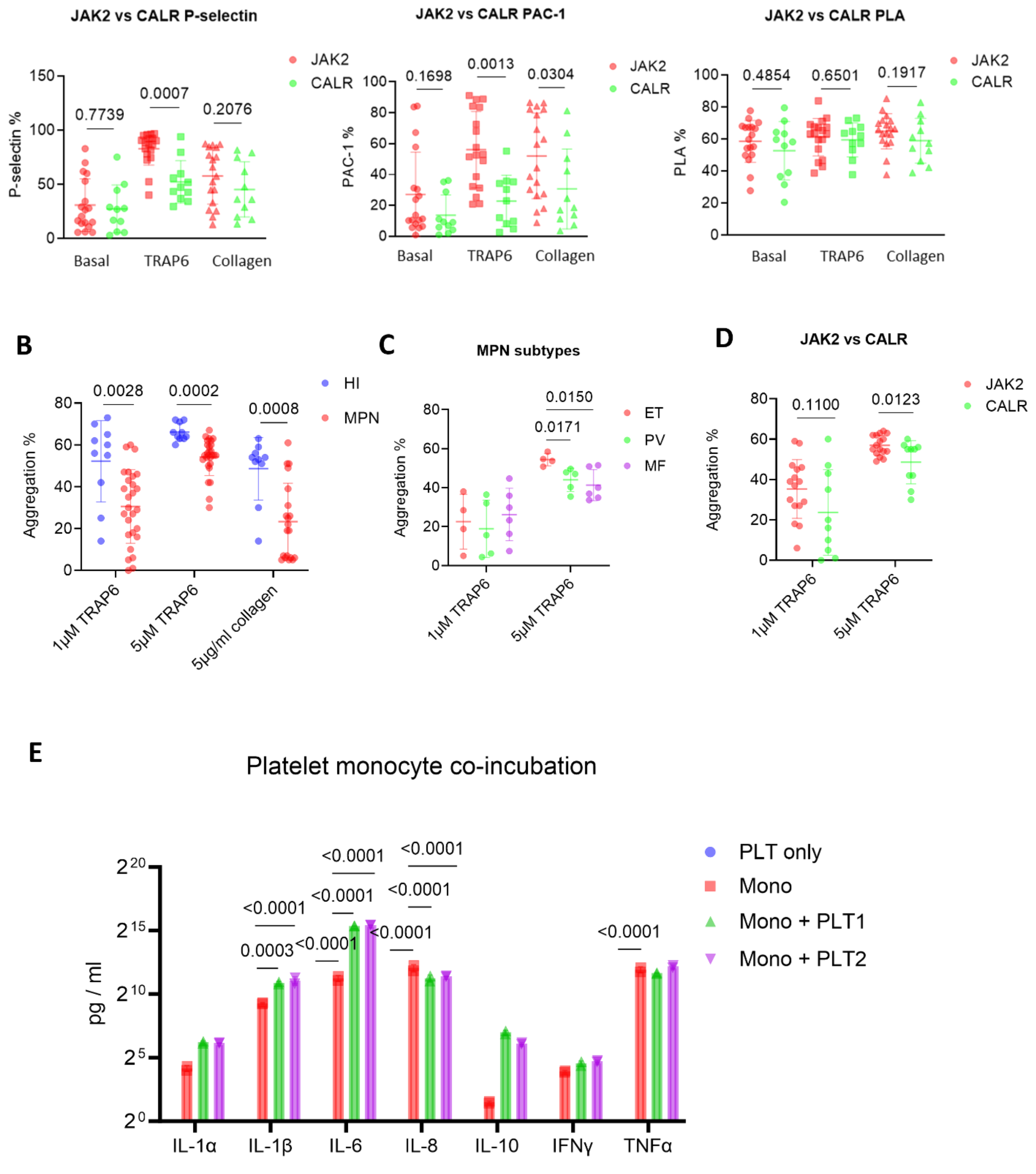

Figure S1. Analysis of platelet activation, secretion and aggregation.

A) Dot plot comparing P-selectin, αIIbβ3 integrin expression and PLA formation of platelets from peripheral blood of ET patients with *JAK2* and *CALR* mutations by flow cytometry. Statistics were assessed by two-tailed Mann-Whitney U test.

B) Dot plot showing maximal aggregation intensity of washed platelets from HI (n = 10) and MPN (n = 27). Data is shown as mean ± SD. Statistics were assessed by two-tailed Student's t test.

C) Dot plot showing maximal aggregation intensity of washed platelets from subjects with different MPN subtypes. Statistics were assessed by two-tailed Student's t test.

D) Dot plot showing maximal aggregation intensity of washed platelets from MPN patients with *JAK2* or *CALR* mutations. Statistics were assessed by two-tailed Student's t test.

E) Bar plot showing cytokine levels in supernatants. Enriched monocytes (0.5M) with or without the incubation of washed platelets (50M) for 6 hours. Supernatants were collected for cytokine determination by multiplex Luminex assay. Statistics were assessed by two-way ANOVA, comparing mean values against the monocytes group. The platelet group are zeros.

Figure S2

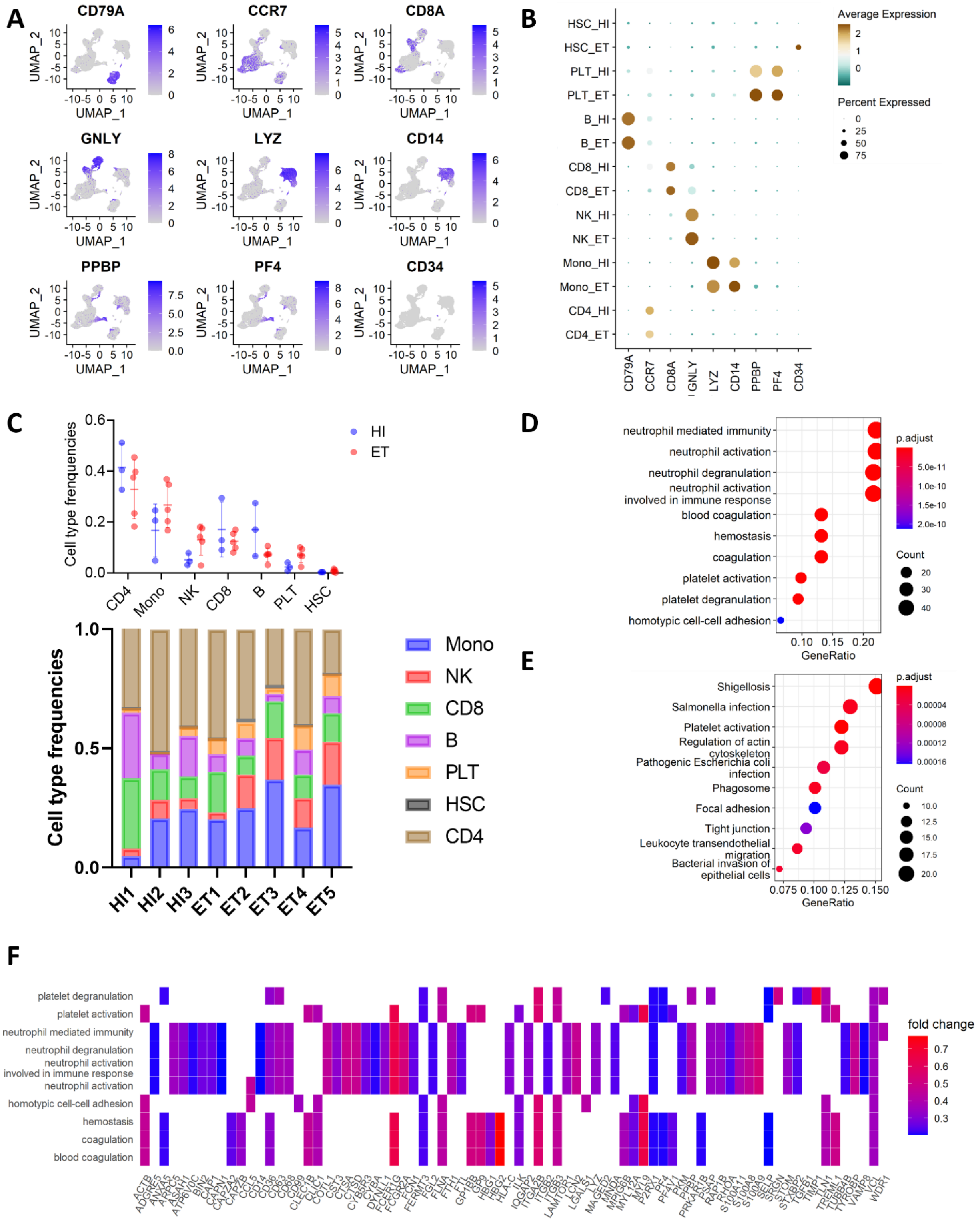
A
CD79A
CCR7
CD8A
GNLY
LYZ
CD14
PPBP
PF4
CD34
UMAP_1
UMAP_2
B
HSC_HI
HSC_ET
PLT_HI
PLT_ET
B_HI
B_ET
CD8_HI
CD8_ET
NK_HI
NK_ET
Mono_HI
Mono_ET
CD4_HI
CD4_ET
Average Expression
Percent Expressed
C
Cell type frenquencies
HI
ET
CD4
Mono
NK
CD8
B
PLT
HSC
Cell type frequencies
HI1
HI2
HI3
ET1
ET2
ET3
ET4
ET5
D
neutrophil mediated immunity
neutrophil activation
neutrophil degranulation
neutrophil activation involved in immune response
blood coagulation
hemostasis
coagulation
platelet activation
platelet degranulation
homotypic cell-cell adhesion
GeneRatio
p.adjust
Count
E
Shigellosis
Salmonella infection
Platelet activation
Regulation of actin cytoskeleton
Pathogenic Escherichia coli infection
Phagosome
Focal adhesion
Tight junction
Leukocyte transendothelial migration
Bacterial invasion of epithelial cells
GeneRatio
p.adjust
Count
F
platelet degranulation
platelet activation
neutrophil mediated immunity
neutrophil degranulation
neutrophil activation involved in immune response
neutrophil activation
homotypic cell-cell adhesion
hemostasis
coagulation
blood coagulation
fold change

Figure S2

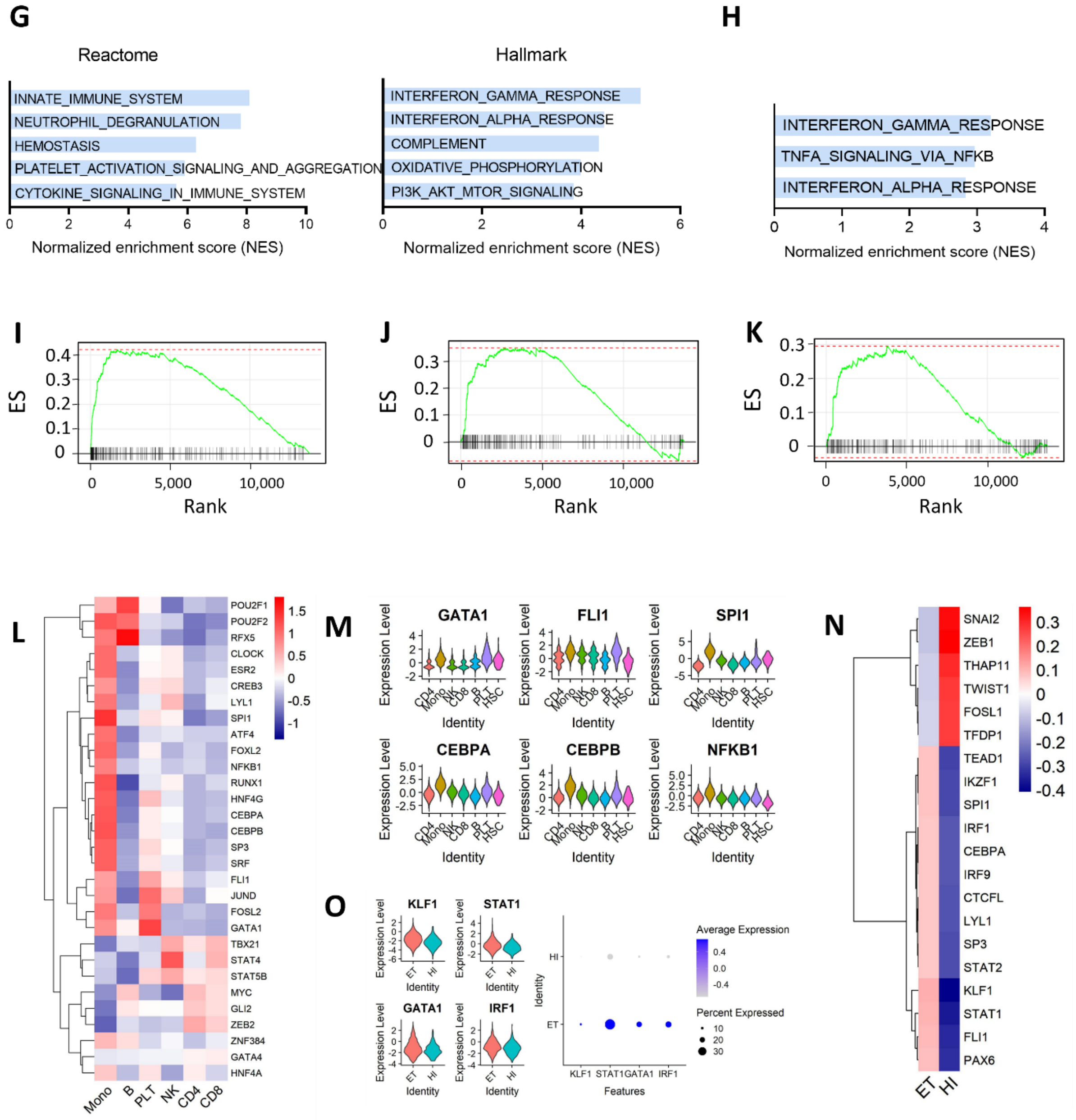

G
Reactome
INNATE_IMMUNE_SYSTEM
NEUTROPHIL_DEGRANULATION
HEMOSTASIS
PLATELET_ACTIVATION_SIGNALING_AND_AGGREGATION
CYTOKINE_SIGNALING_IN_IMMUNE_SYSTEM
Normalized enrichment score (NES)
Hallmark
INTERFERON_GAMMA_RESPONSE
INTERFERON_ALPHA_RESPONSE
COMPLEMENT
OXIDATIVE_PHOSPHORYLATION
PI3K_AKT_MTOR_SIGNALING
Normalized enrichment score (NES)
H
INTERFERON_GAMMA_RESPONSE
TNFA_SIGNALING_VIA_NFKB
INTERFERON_ALPHA_RESPONSE
Normalized enrichment score (NES)
I
J
K
ES
Rank
L
POU2F1
POU2F2
RFX5
CLOCK
ESR2
CREB3
LYL1
SPI1
ATF4
FOXL2
NFKB1
RUNX1
HNF4G
CEBPA
CEBPB
SP3
SRF
FLI1
JUND
FOSL2
GATA1
TBX21
STAT4
STAT5B
MYC
GLI2
ZEB2
ZNF384
GATA4
HNF4A
Mono
B
PLT
NK
CD4
CD8
M
GATA1
FLI1
SPI1
CEBPA
CEBPB
NFKB1
Expression Level
Identity
O
KLF1
STAT1
GATA1
IRF1
ET
HI
Average Expression
Percent Expressed
Features
N
SNAI2
ZEB1
THAP11
TWIST1
FOSL1
TFDP1
TEAD1
IKZF1
SPI1
IRF1
CEBPA
IRF9
CTCFL
LYL1
SP3
STAT2
KLF1
STAT1
FLI1
PAX6
ET
HI

Figure S2. scRNA-seq analysis.

A) Feature plots of genes for cell type identification.

B) Dot plot of top differentially expressed genes for cell type identification.

C) Dot plot and stacked bar plot showing frequency of different type of cells in all 8 samples for scRNA-seq.

D) GO enrichment analysis of differentially-expressed genes (DEGs) in platelets from ET vs. HI.

E) KEGG enrichment analysis of DEGs in platelets from ET vs. HI.

F) Heatmap showing the distribution of DEGs in GO enrichment pathways.

G) Bar plot showing top 5 reactome and hallmark pathways enriched in platelets from ET vs. HI.

H) Bar plot showing top 3 hallmark pathways enriched in platelets from ET carrying *JAK2* mutations vs. ET carrying *CALR* mutations.

I) GSEA enrichment plots for “reactome platelet activation signaling and aggregation” gene set enriched in platelets from ET vs. HI.

J) GSEA enrichment plots for “hallmark IFN-γ response” gene set enriched in platelets from ET vs. HI.

K) GSEA enrichment plots for “hallmark OXPHOS” gene set enriched in platelets from ET vs. HI.

L) Heatmap showing top 20 most variable transcriptional factors (TFs) in each type of cells based on VIPER scores on DoRothEA’s regulons.

M) Violin plots showing the expression of representative TFs in each type of cells.

N) Heatmap showing top 20 most variable TFs in platelets from ET vs. HI.

O) Violin plots and dot plots showing the expression of representative TFs in platelets from ET and HI.

Figure S3

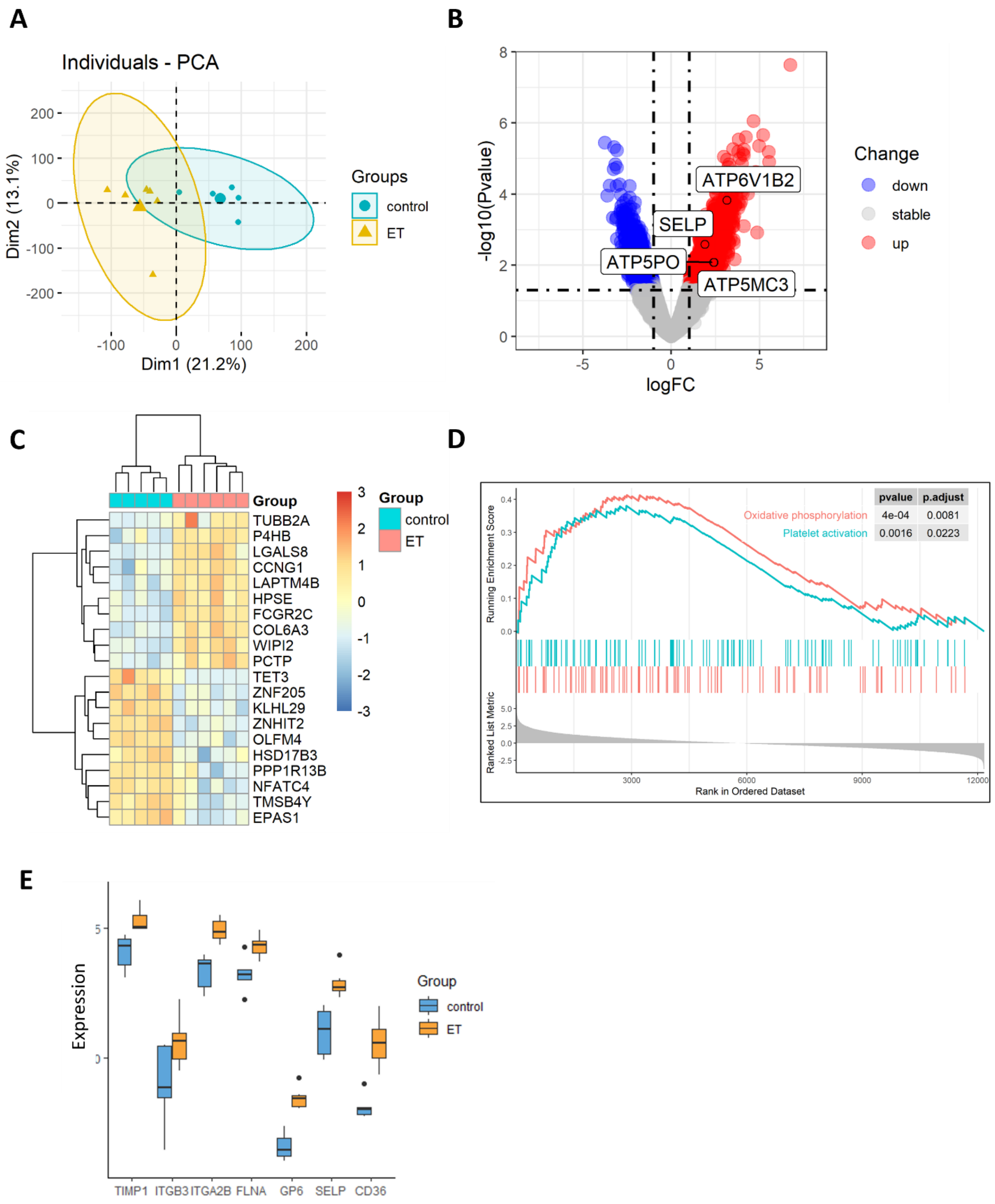
A
Individuals - PCA
Dim2 (13.1%)
Dim1 (21.2%)
Groups
control
ET
B
-log10(Pvalue)
logFC
ATP6V1B2
SELP
ATP5PO
ATP5MC3
Change
down
stable
up
C
Group
TUBB2A
P4HB
LGALS8
CCNG1
LAPTM4B
HPSE
FCGR2C
COL6A3
WIPI2
PCTP
TET3
ZNF205
KLHL29
ZNHIT2
OLFM4
HSD17B3
PPP1R13B
NFATC4
TMSB4Y
EPAS1
Group
control
ET
D
pvalue
p.adjust
Oxidative phosphorylation
4e-04
0.0081
Platelet activation
0.0016
0.0223
Running Enrichment Score
Ranked List Metric
Rank in Ordered Dataset
E
Expression
TIMP1
ITGB3
ITGA2B
FLNA
GP6
SELP
CD36
Group
control
ET

Figure S3. Analysis of GSE2006 microarray between platelets from HIs and ET patients.

A) PCA plot of platelets from ET (n = 6) and HI (n = 5) in GSE2006.

B) Volcano plot showing differentially expressed genes (DEG) between platelets from ET and HI in GSE2006.

C) Heatmap of top 10 DEGs in HI and ET in GSE2006.

D) GSEA enrichment plots for "hallmark OXPHOS" and "reactome platelet activation signaling and aggregation" gene sets enriched in platelets from ET.

E) Bar plot showing expression of representative genes in GSE2006 (|fold change| >2 & P <0.05).

Figure S4

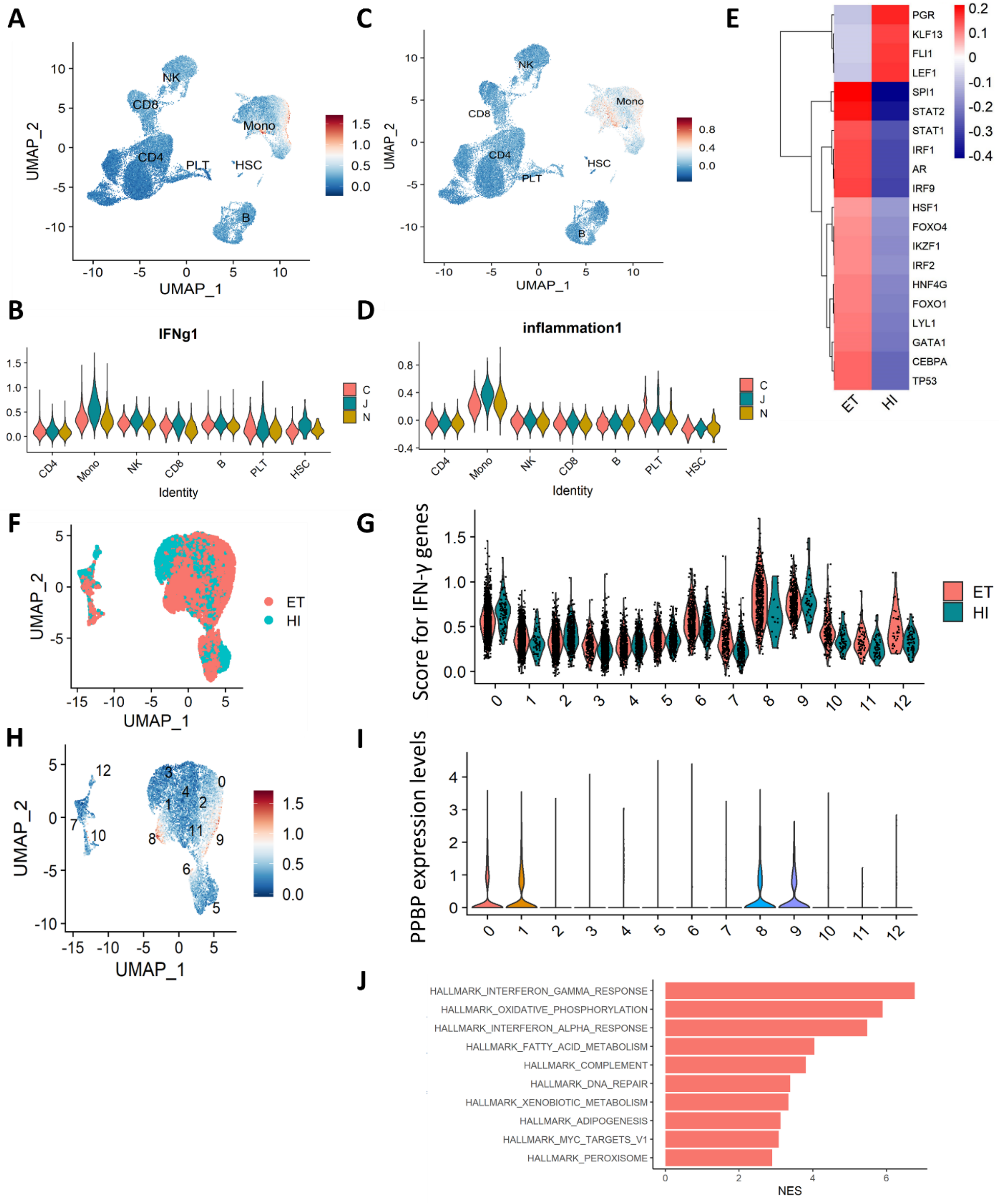
A
C
E
B
D
F
G
H
I
J
UMAP_1
UMAP_2
NK
CD8
CD4
PLT
HSC
Mono
B
IFNg1
inflammation1
Identity
C
J
N
PGR
KLF13
FLI1
LEF1
SPI1
STAT2
STAT1
IRF1
AR
IRF9
HSF1
FOXO4
IKZF1
IRF2
HNF4G
FOXO1
LYL1
GATA1
CEBPA
TP53
ET
HI
Score for IFN-γ genes
PPBP expression levels
HALLMARK_INTERFERON_GAMMA_RESPONSE
HALLMARK_OXIDATIVE_PHOSPHORYLATION
HALLMARK_INTERFERON_ALPHA_RESPONSE
HALLMARK_FATTY_ACID_METABOLISM
HALLMARK_COMPLEMENT
HALLMARK_DNA_REPAIR
HALLMARK_XENOBIOTIC_METABOLISM
HALLMARK_ADIPOGENESIS
HALLMARK_MYC_TARGETS_V1
HALLMARK_PEROXISOME
NES

Figure S4. scRNA-seq analysis of monocytes.

A) UMAP plot showing scores for genes in “hallmark interferon gamma response” gene set.

B) Violin plot showing scores for genes in “hallmark interferon gamma response” gene set (C: ET with CALR mutations; J: ET with JAK2 mutation; N: HI).

C) UMAP plot showing scores for genes in “hallmark inflammatory response” gene set.

D) Violin plot showing scores for genes in “hallmark inflammatory response” gene set (C: ET with CALR mutations; J: ET with JAK2 mutation; N: HI).

E) Heatmap showing top 20 most variable TFs in monocytes from ET vs. HI.

F) UMAP plot showing scores for genes in “hallmark interferon gamma response” gene set in monocytes from HI and ET.

G) Violin plot showing scores for genes in “hallmark interferon gamma response” gene set in monocytes from HI and ET.

H) UMAP plot showing PPBP (a marker for platelets) expression levels in monocytes from HI and ET.

I) Violin plots showing PPBP (a marker for platelets) expression levels in monocytes from HI and ET.

J) Bar plot showing top 10 hallmark pathways enriched in monocytes from ET vs. HI.

Figure S5

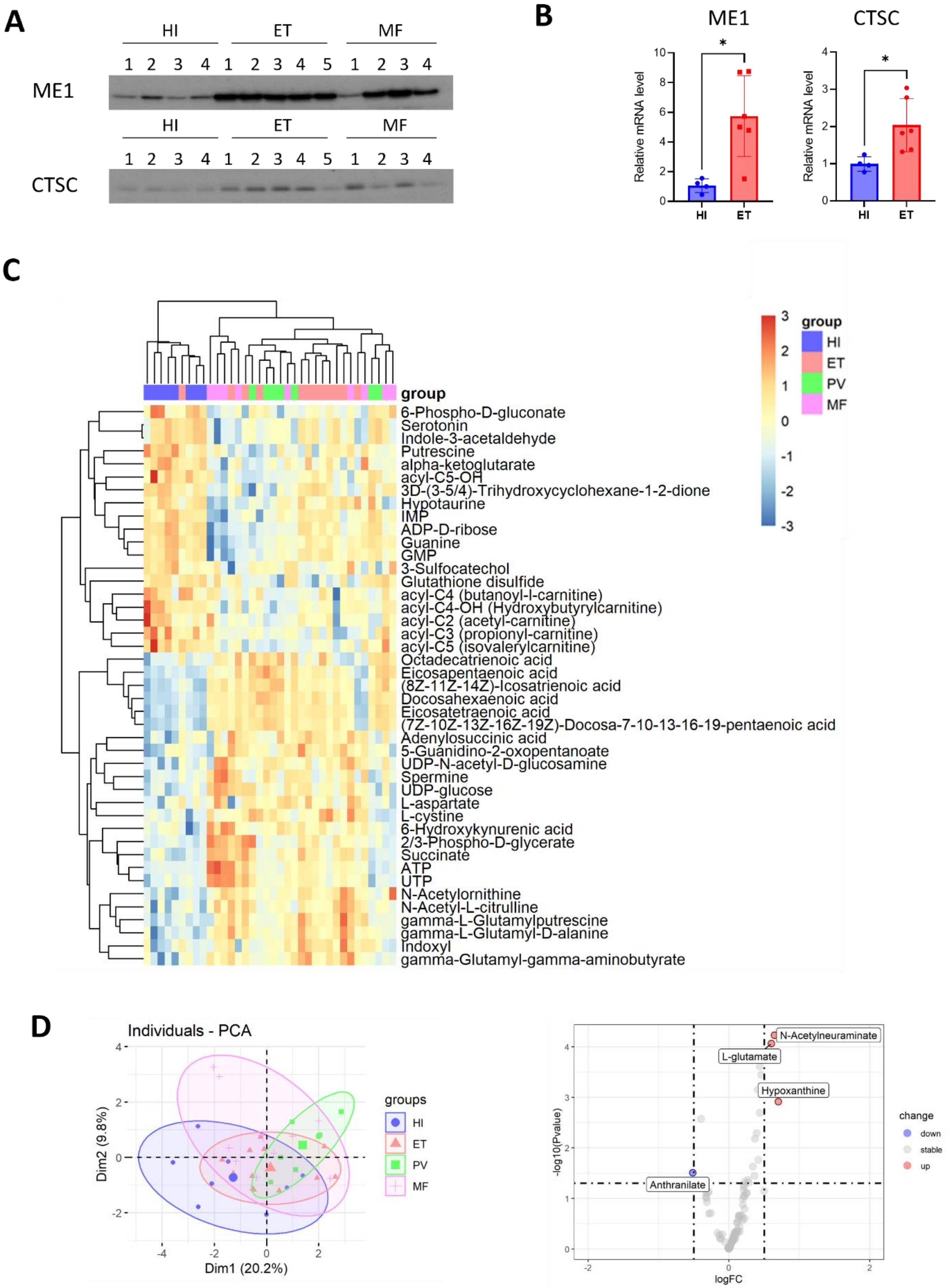
A
HI
ET
MF
1 2 3 4 1 2 3 4 5 1 2 3 4
ME1
CTSC
B
ME1
CTSC
Relative mRNA level
HI
ET
*
C
group
HI
ET
PV
MF
6-Phospho-D-gluconate
Serotonin
Indole-3-acetaldehyde
Putrescine
alpha-ketoglutarate
acyl-C5-OH
3D-(3-5/4)-Trihydroxycyclohexane-1-2-dione
Hypotaurine
IMP
ADP-D-ribose
Guanine
GMP
3-Sulfocatechol
Glutathione disulfide
acyl-C4 (butanoyl-l-carnitine)
acyl-C4-OH (Hydroxybutyrylcarnitine)
acyl-C2 (acetyl-carnitine)
acyl-C3 (propionyl-carnitine)
acyl-C5 (isovalerylcarnitine)
Octadecatrienoic acid
Eicosapentaenoic acid
(8Z-11Z-14Z)-Icosatrienoic acid
Docosahexaenoic acid
Eicosatetraenoic acid
(7Z-10Z-13Z-16Z-19Z)-Docosa-7-10-13-16-19-pentaenoic acid
Adenylosuccinic acid
5-Guanidino-2-oxopentanoate
UDP-N-acetyl-D-glucosamine
Spermine
UDP-glucose
L-aspartate
L-cystine
6-Hydroxykynurenic acid
2/3-Phospho-D-glycerate
Succinate
ATP
UTP
N-Acetylornithine
N-Acetyl-L-citrulline
gamma-L-Glutamylputrescine
gamma-L-Glutamyl-D-alanine
Indoxyl
gamma-Glutamyl-gamma-aminobutyrate
D
Individuals - PCA
Dim1 (20.2%)
Dim2 (9.8%)
groups
HI
ET
PV
MF
N-Acetylneuraminate
L-glutamate
Hypoxanthine
Anthranilate
-log10(Pvalue)
logFC
change
down
stable
up

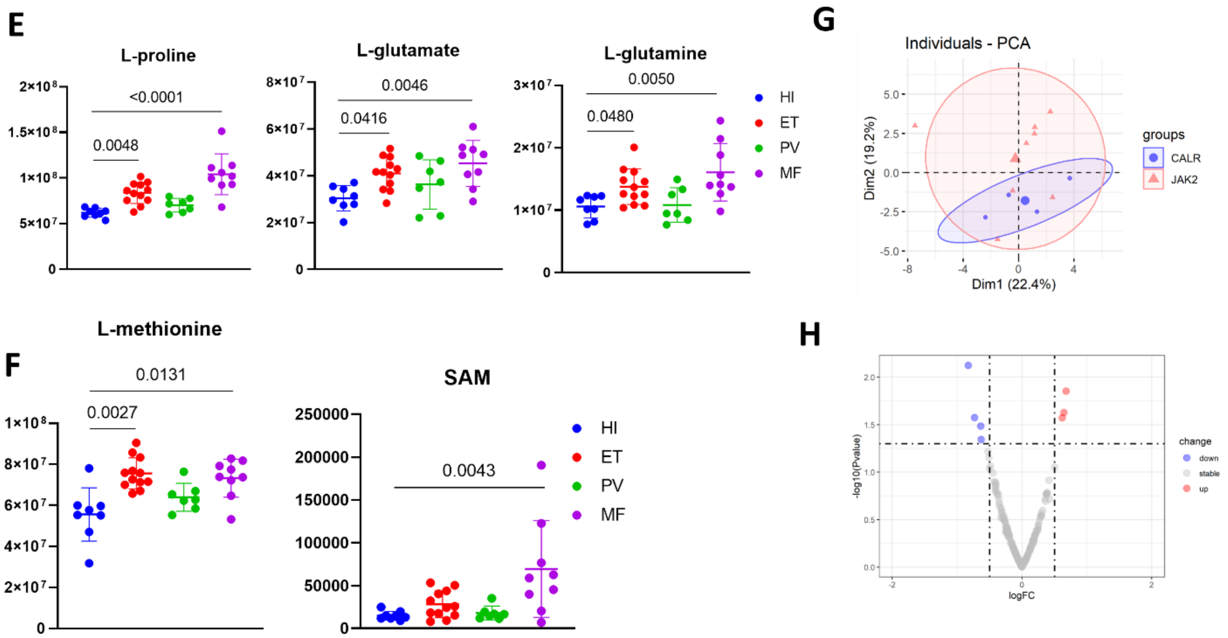
E
L-proline
2×10⁸
1.5×10⁸
1×10⁸
5×10⁷
0
<0.0001
0.0048
L-glutamate
8×10⁷
6×10⁷
4×10⁷
2×10⁷
0
0.0046
0.0416
L-glutamine
3×10⁷
2×10⁷
1×10⁷
0
0.0050
0.0480
HI
ET
PV
MF
G
Individuals - PCA
Dim2 (19.2%)
Dim1 (22.4%)
5.0
2.5
0.0
-2.5
-5.0
-8
-4
0
4
groups
CALR
JAK2
L-methionine
F
1×10⁸
8×10⁷
6×10⁷
4×10⁷
2×10⁷
0
0.0131
0.0027
SAM
250000
200000
150000
100000
50000
0
0.0043
HI
ET
PV
MF
H
-log10(Pvalue)
logFC
change
down
stable
up

Figure S5. Platelet and plasma proteomics and metabolomics analysis.

A) Protein levels of ME1 and CTSC in washed platelets from HI and MPN.

B) Relative gene expression of ME1 and CTSC in washed platelets from HI and MPN.

C) Heatmap of down- (n = 19) and up-regulated (n = 24) metabolites in platelets between ET and HI (P<0.05, |fold change| > 2^0.5).

D) PCA plot and volcano plot of plasma DAMs between MPN and HI.

E) Dot plots of peak areas (arbitrary units after normalization) for proline, glutamate and glutamine of platelet. Statistics were assessed by one-way ANOVA. P values are marked if <0.05.

F) Dot plots of peak areas (arbitrary units after normalization) for methionine and SAM of platelet. Statistics were assessed by one-way ANOVA. P values are marked if <0.05.

G) PCA scores plot of metabolites LC-MS data of platelets from ET (JAK = 8, CALR = 4) displayed with 80% confidence region.

H) Volcano plot of metabolite changes in ET carrying JAK2 (n = 8) vs. ET carrying CALR (n = 4) mutations. Red dots denote significant (p < 0.05) and fold change (> 2^0.5) features, blue dots denote significant (p < 0.05) and fold change (< -2^0.5) features.

Figure S6

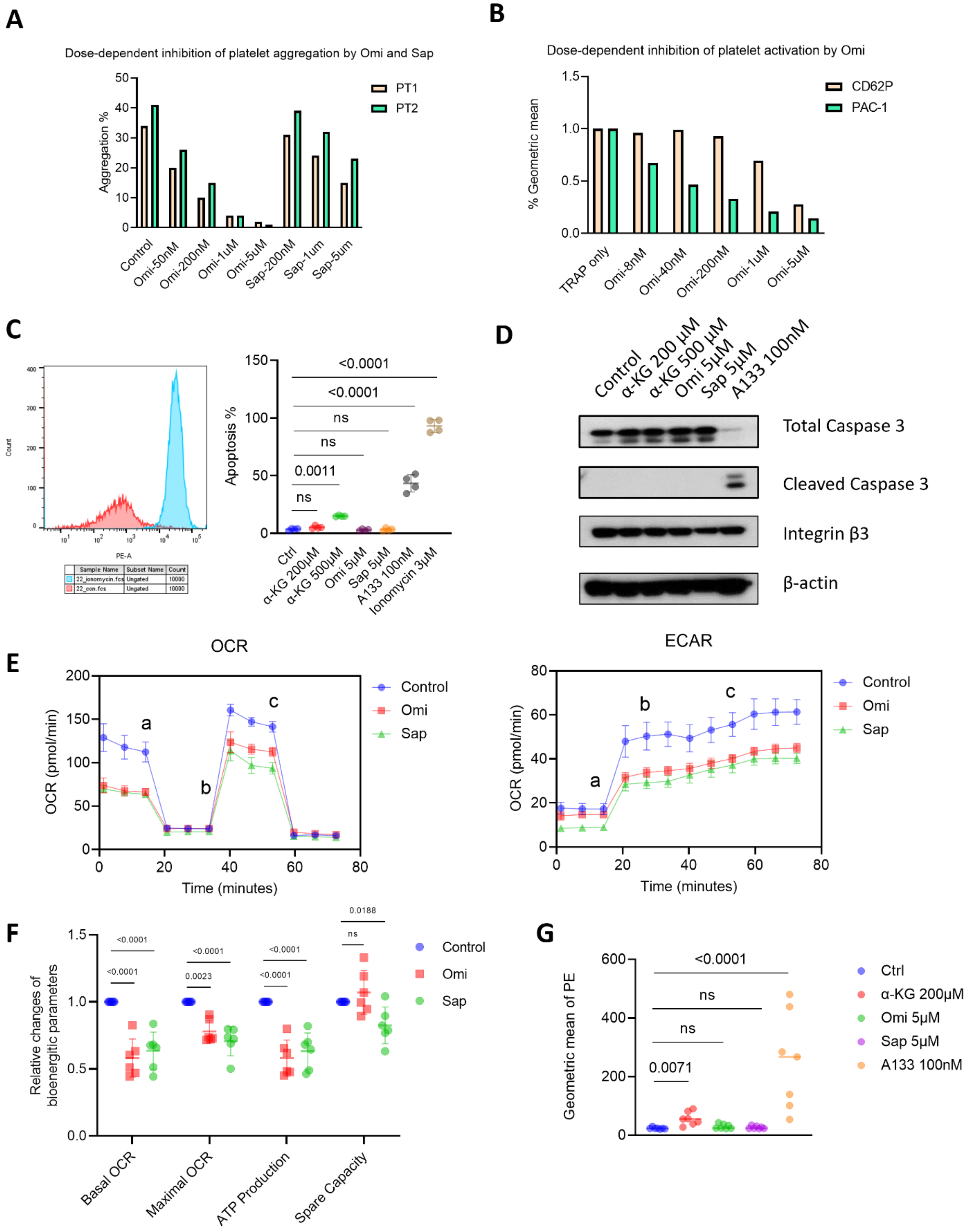
A
Dose-dependent inhibition of platelet aggregation by Omi and Sap
Aggregation %
PT1
PT2
Control
Omi-50nM
Omi-200nM
Omi-1uM
Omi-5uM
Sap-200nM
Sap-1um
Sap-5um
B
Dose-dependent inhibition of platelet activation by Omi
% Geometric mean
CD62P
PAC-1
TRAP only
Omi-8nM
Omi-40nM
Omi-200nM
Omi-1uM
Omi-5uM
C
Count
PE-A
Sample Name
Subset Name
Count
22_ionomycin.fcs
Ungated
10000
22_con.fcs
Ungated
10000
Apoptosis %
<0.0001
<0.0001
ns
ns
0.0011
ns
Ctrl
α-KG 200μM
α-KG 500μM
Omi 5μM
Sap 5μM
A133 100nM
Ionomycin 3μM
D
Control
α-KG 200 μM
α-KG 500 μM
Omi 5μM
Sap 5μM
A133 100nM
Total Caspase 3
Cleaved Caspase 3
Integrin β3
β-actin
E
OCR
OCR (pmol/min)
Time (minutes)
Control
Omi
Sap
a
b
c
ECAR
OCR (pmol/min)
Time (minutes)
Control
Omi
Sap
a
b
c
F
Relative changes of bioenergitic parameters
<0.0001
<0.0001
<0.0001
0.0023
<0.0001
<0.0001
0.0188
ns
Control
Omi
Sap
Basal OCR
Maximal OCR
ATP Production
Spare Capacity
G
Geometric mean of PE
<0.0001
ns
ns
0.0071
Ctrl
α-KG 200μM
Omi 5μM
Sap 5μM
A133 100nM

Figure S6. Effects of mTOR inhibitors and α-KG on platelets and cell lines.

A) Bar plot showing the effects of omipalisib or sapanisertib on MPN platelet aggregation. Washed platelets were incubated with omipalisib or sapanisertib for one hour followed by platelet aggregation analysis with 5 μM TRAP6 stimulation.

B) Bar plot showing the effects of omipalisib on MPN platelet activation. Washed platelets were incubated with omipalisib for one hour followed by 1 μM TRAP6 stimulation and flow cytometry analysis.

C) Representative image and dot plot showing the induction of apoptosis of washed MPN platelets. Washed platelets were treated with reagents as shown for one hour followed by annexin V apoptosis assay and flow cytometry analysis. Statistics were assessed by one-way ANOVA.

D) Immunoblots of washed platelets showing the induction of apoptosis. Washed MPN platelets were incubated with different treatments for one hour followed by immunoblots.

E) Representative OCR and ECAR profiles of MPN platelets (a: Oligomycin A; b: FCCP; c: Rotenone/antimycin A).

F) Quantification of basal OCR, ATP production, maximal OCR and spare capacity profiles of washed MPN platelets (n = 6). Data were normalized to DMSO group set as 1. Data are mean ± SD. Statistics were assessed by two-way ANOVA.

G) Dot plot showing geometric mean fluorescence intensity of washed MPN platelets after different treatments for one hour followed by MitoSOX-based flow cytometry analysis (n = 7). Statistics were assessed by paired one-way ANOVA.

Figure S7

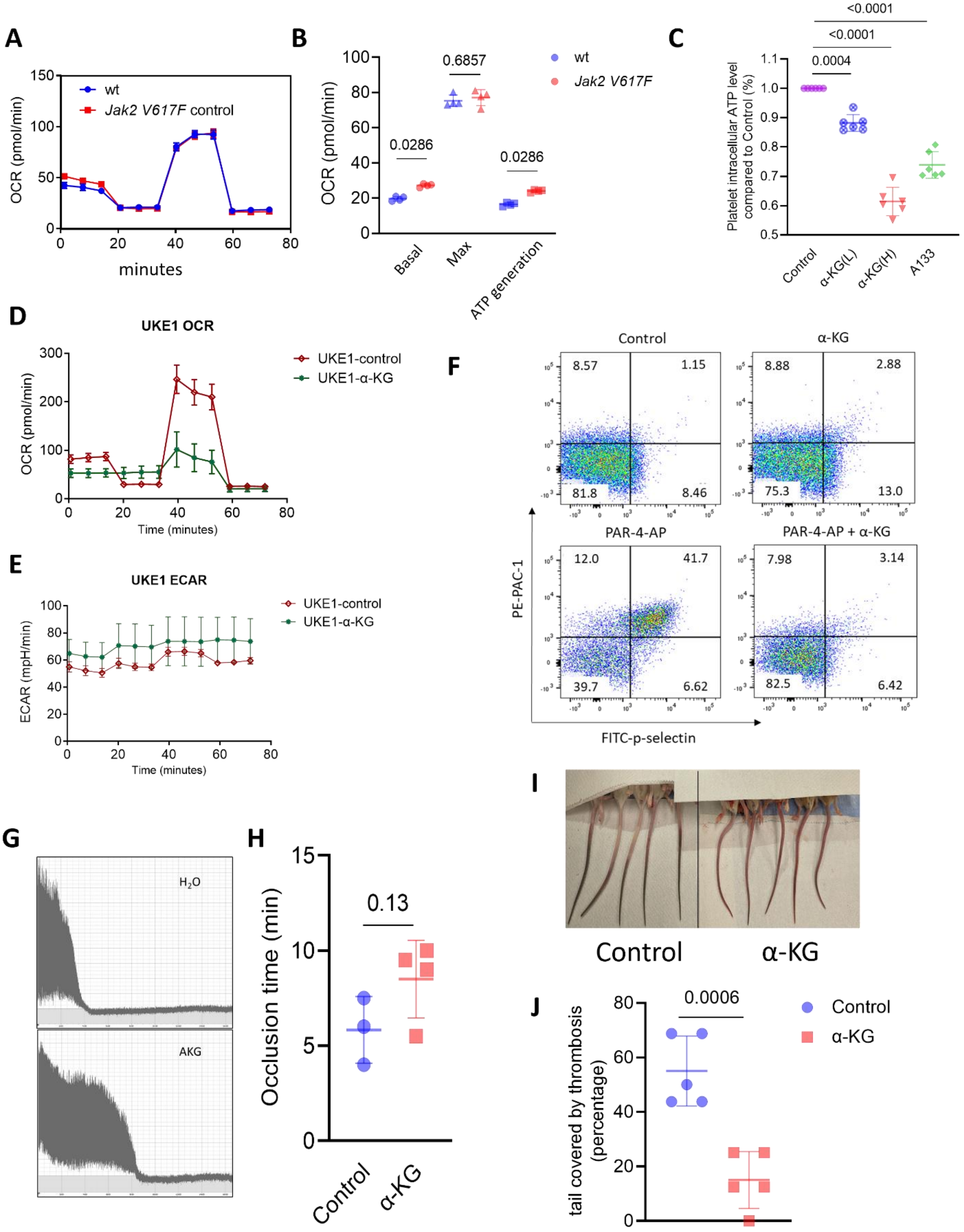

A
OCR (pmol/min)
minutes
wt
Jak2 V617F control
B
OCR (pmol/min)
wt
Jak2 V617F
0.0286
0.6857
0.0286
Basal
Max
ATP generation
C
Platelet intracellular ATP level compared to Control (%)
<0.0001
<0.0001
0.0004
Control
α-KG(L)
α-KG(H)
A133
D
UKE1 OCR
OCR (pmol/min)
Time (minutes)
UKE1-control
UKE1-α-KG
E
UKE1 ECAR
ECAR (mpH/min)
Time (minutes)
UKE1-control
UKE1-α-KG
F
Control
α-KG
8.57
1.15
81.8
8.46
8.88
2.88
75.3
13.0
PAR-4-AP
PAR-4-AP + α-KG
12.0
41.7
39.7
6.62
7.98
3.14
82.5
6.42
PE-PAC-1
FITC-p-selectin
G
H_2O
AKG
H
Occlusion time (min)
0.13
Control
α-KG
I
Control
α-KG
J
tail covered by thrombosis (percentage)
0.0006
Control
α-KG

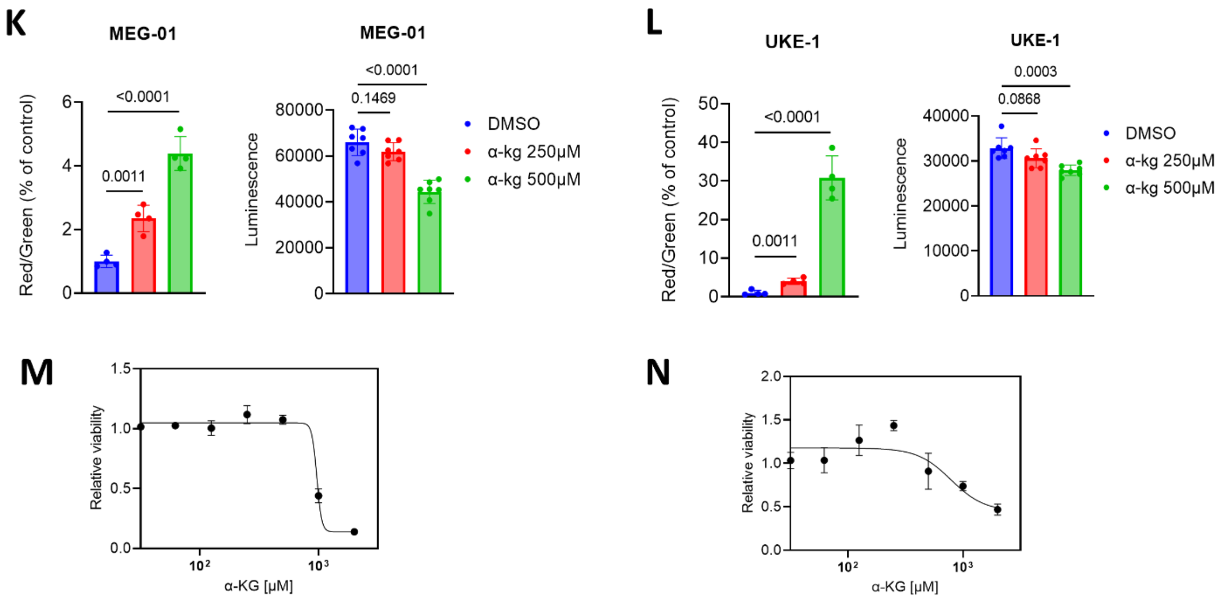
K
MEG-01
Red/Green (% of control)
<0.0001
0.0011
MEG-01
Luminescence
<0.0001
0.1469
DMSO
α-kg 250μM
α-kg 500μM
L
UKE-1
Red/Green (% of control)
<0.0001
0.0011
UKE-1
Luminescence
0.0003
0.0868
DMSO
α-kg 250μM
α-kg 500μM
M
Relative viability
α-KG [μM]
N
Relative viability
α-KG [μM]

Figure S7. Effects of α-KG on mouse platelets, cell lines and thrombosis mouse models.

A) Representative OCR profiles of platelets from wild type and *Jak2 V617F* knock-in mice.

B) Quantification of mitochondrial respiration parameters (n = 4) of platelets from wild type and *Jak2 V617F* knock-in mice. Data are mean ± SD. Statistics were assessed by two-tailed Mann-Whitney U test.

C) Quantification of intracellular ATP level changes of washed MPN platelets after different treatments for one hour (n = 6). Data were normalized to control group set as 1. Data are mean ± SD. Statistics were assessed by one-way ANOVA.

D) Representative OCR profiles of UKE-1 cells with the incubation of Octyl-α-KG or DMSO.

E) Representative ECAR profiles of UKE-1 cells with the incubation of Octyl-α-KG or DMSO.

F) Representative images showing effects of α-KG on activation of washed platelets from *Jak2 V617F* knock-in mice. Washed platelets from *Jak2 V617F* knock-in mice were treated with Octyl-α-KG for one hour followed by PAR-4-AP stimulation and flow cytometry analysis.

G) Representative image showing blood flow in the carotid artery following FeCl3-induced injury until complete vessel occlusion. *Jak2 V617F* mice were fed with either regular water (n = 3) or 2% α-KG (n = 4) water for 12 days before FeCl3-induced vascular injury.

H) Statistical analysis of vessel occlusion time. Data are mean ± SD. Statistics were assessed by two-tailed Student's t test.

I) Tail thrombosis images. BALB/c mice were fed with either regular water (n = 5) or 2% α-KG (n = 5) water for 10 days before intraperitoneal injection of 100 μl carrageenan (10mg/ml). Tail thrombosis were observed 48 hours after carrageenan injection.

J) Statistical analysis of tail thrombosis percentage. Data are mean ± SD. Statistics were assessed by two-tailed Student's t test.

K) Bar plot showing the effects of α-KG on mitochondrial membrane potential (left) and intracellular ATP level (right) in MEG-01 cells determined by JC-1 dye staining and ATPlite luminescence assay.

L) Bar plot showing the effects of α-KG on mitochondrial membrane potential (left) and intracellular ATP level (right) in UKE-1 cells determined by JC-1 dye staining and ATPlite luminescence assay.

M) Cell viability curve showing the effects of α-KG on the proliferation of MEG-01 cells after 72 hours (IC50 = 967.5μM).

N) Cell viability curve showing the effects of α-KG on the proliferation of UKE-1 cells after 72 hours (IC50 = 785.6μM).

Figure S8

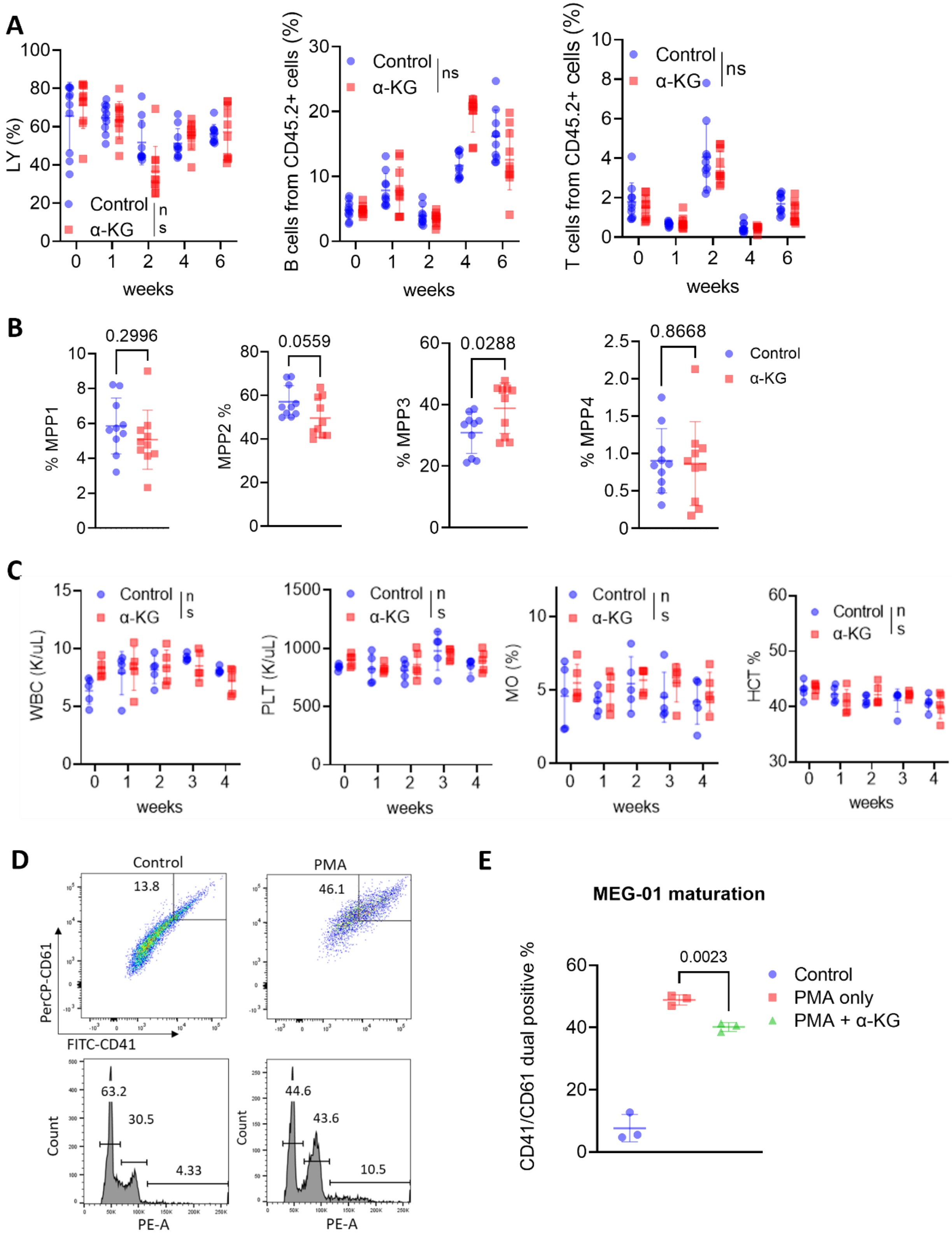
A
LY (%)
Control
α-KG
ns
weeks
B cells from CD45.2+ cells (%)
T cells from CD45.2+ cells (%)
B
0.2996
% MPP1
0.0559
MPP2 %
0.0288
% MPP3
0.8668
% MPP4
C
WBC (K/uL)
PLT (K/uL)
MO (%)
HCT %
D
Control
PMA
13.8
46.1
PerCP-CD61
FITC-CD41
63.2
30.5
4.33
44.6
43.6
10.5
Count
PE-A
E
MEG-01 maturation
0.0023
CD41/CD61 dual positive %
PMA only
PMA + α-KG

Figure S8. *in vivo* treatment of α-KG inhibited MPN progression.

A) Lymphocytes (LY), B and T cell ratios from *Jak2 V617F* transplanted mice treated with vehicle or α-KG across multiple timepoints. Data are mean ± SD. Statistics were assessed by two-way ANOVA with Dunnett's multiple comparisons test (ns: not significant, $P > 0.05$).

B) Percentage of MPPs from *Jak2 V617F* transplanted mice treated with vehicle or α-KG. Data are mean ± SD. Statistics were assessed by two-tailed Student's t test.

C) WBC, platelet counts, monocytes and HCT ratios from wild type C57BL/6J mice treated with vehicle (n = 5) or α-KG (n = 5) across multiple timepoints. Data are mean ± SD. Statistics were assessed by two-way ANOVA with Dunnett's multiple comparisons test (ns: not significant, $P > 0.05$).

D) Representative images showing the maturation of MEG-01 cells with PMA treatment (CD41/CD61 expression and ploidy analysis by flow cytometry)

E) Quantification of percentages of CD41/CD61 dual positive cells with α-KG treatment. Data are mean ± SD. Statistics were assessed by two-tailed Student's t test. MEG-01 cells were treated with PMA for 2 days with 500 μM Octyl-α-KG or DMSO control. The ratio of CD41- and CD61-dual positive cells were determined by flow cytometry (n =3). Ploidy analysis was determined by propidium iodide (PI) staining.

Figure S9

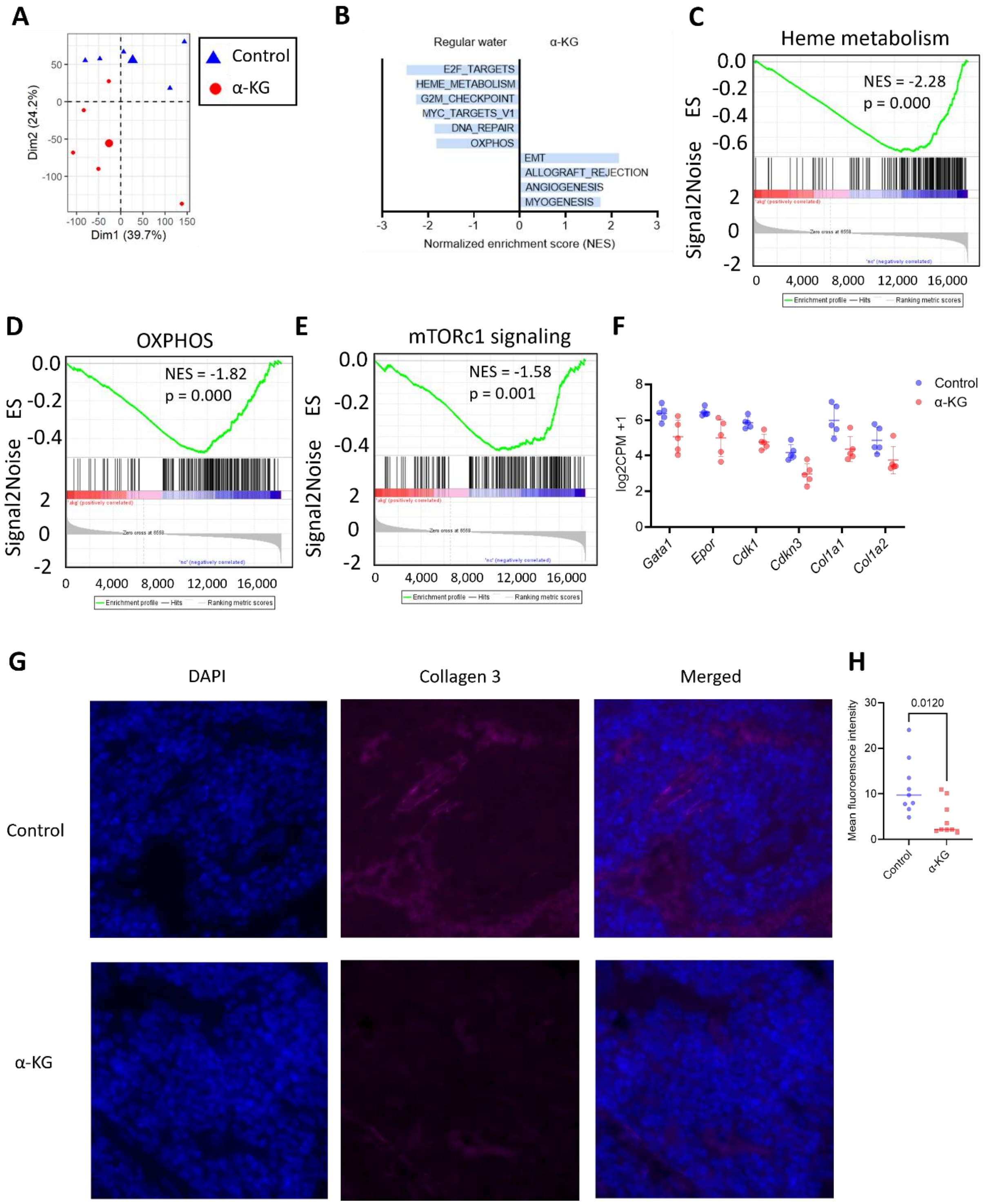
A
Control
α-KG
Dim2 (24.2%)
Dim1 (39.7%)
B
Regular water
α-KG
E2F_TARGETS
HEME_METABOLISM
G2M_CHECKPOINT
MYC_TARGETS_V1
DNA_REPAIR
OXPHOS
EMT
ALLOGRAFT_REJECTION
ANGIOGENESIS
MYOGENESIS
Normalized enrichment score (NES)
C
Heme metabolism
NES = -2.28
p = 0.000
ES
Signal2Noise
Enrichment profile
Hits
Ranking metric scores
D
OXPHOS
NES = -1.82
p = 0.000
E
mTORc1 signaling
NES = -1.58
p = 0.001
F
Control
α-KG
log2CPM +1
Gata1
Epor
Cdk1
Cdkn3
Col1a1
Col1a2
G
DAPI
Collagen 3
Merged
Control
α-KG
H
0.0120
Mean fluoroensnce intensity
Control
α-KG

Figure S9. Bulk RNA-seq of bone marrow from control (n = 5) and 1% α-KG-supplemented *Jak2 V617F* transplanted mice (n = 5) and collagen staining.

A) PCA plot for bulk RNA-seq data of bone marrow from control and α-KG-supplemented mice.

B) Bar plot of top 10 hallmark pathways with the highest GSEA enrichment scores when comparing between control and α-KG-supplemented groups.

C) GSEA enrichment plots for “Oxidative phosphorylation” gene set enriched in control vs. α-KG-supplemented mice.

D) GSEA enrichment plots for “Heme metabolism” gene set enriched in control vs. α-KG-supplemented mice.

E) GSEA enrichment plots for “mTORc1 signaling” gene set enriched in control vs. α-KG-supplemented mice.

F) Dot plot of RNA-seq counts of representative genes (|fold change| > 2^0.5 & P < 0.05).

G) Representative immunofluorescence images (X100) of femur sections stained for collagen III (magenta); nuclei were stained blue with DAPI. Control and α-KG-supplemented mice were analyzed 6 weeks after α-KG supplementation.

H) Normalized fluorescence intensity for collagen III. Data are mean ± SD. Statistics were assessed by Welch’s t test.

Figure S10

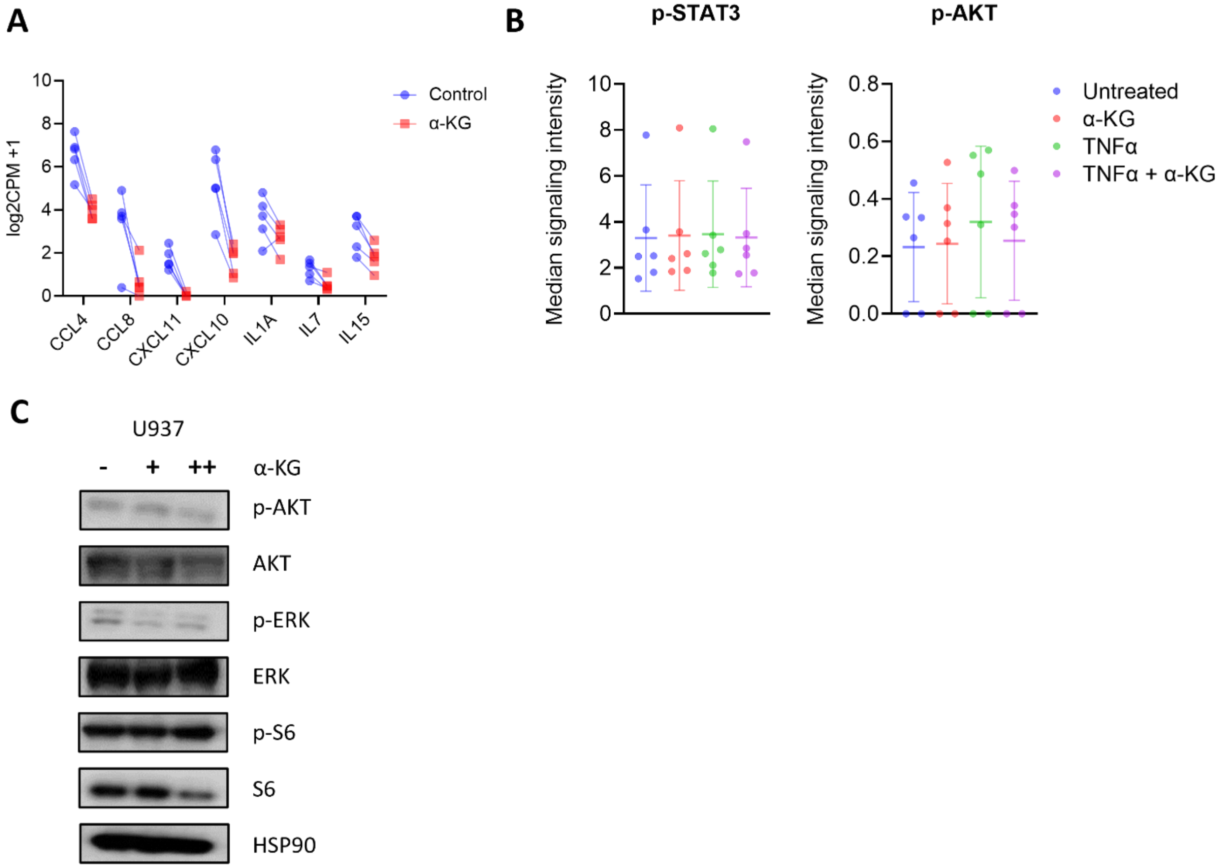
A
10
8
6
4
2
0
log2CPM +1
CCL4
CCL8
CXCL11
CXCL10
IL1A
IL7
IL15
Control
α-KG
B
p-STAT3
p-AKT
Median signaling intensity
10
8
6
4
2
0
Median signaling intensity
0.8
0.6
0.4
0.2
0.0
Untreated
α-KG
TNFα
TNFα + α-KG
C
U937
-
+
++
α-KG
p-AKT
AKT
p-ERK
ERK
p-S6
S6
HSP90

Figure S10. Effects of α-KG on monocyte gene expression and signaling pathways.

A) Dot plot of RNA-seq counts of representative chemokine and cytokine genes in CD14+ monocytes (fold change < -2, $P < 0.05$).

B) Dot plots of intracellular pathways of monocytes in peripheral blood of MPN patients by mass cytometry. Whole blood from MPN patients ($n = 6$) were incubated with Octyl-α-KG or DMSO for 1 hour followed by the stimulation of TNFα. Samples were processed for the determination of intracellular pathway activities by CyTOF. Data are mean ± SD.

C) Immunoblots of U937 cells after α-KG treatment. U937 cells were incubated with 250 μM or 500 μM Octyl-α-KG for one hour followed by immunoblot analysis.

Supplementary methods

Measurement of apoptosis markers in platelets

Surface exposure of phosphatidylserine was assessed in washed platelets by measuring the binding of PE-labelled annexin V (BD Biosciences). Washed platelets were resuspended in annexin V binding buffer (10 mM HEPES, 10 mM sodium hydroxide, 140 mM sodium chloride, 2.5 mM calcium chloride, pH 7.4), incubated with different reagents for 1 hour followed by annexin V staining for 15 minutes at room temperature in the dark. Positive control group was incubated with 3 μM ionomycin during annexin V staining. The samples were analyzed by flow cytometry.

Washed platelets were resuspended in Modified Tyrode's buffer. After being incubated with different reagents for 1 hour, platelets were pelleted and lysed for immunoblot analysis of caspase-3 cleavage.

Determination of intracellular ATP level

Intracellular ATP levels were determined by ATPlite Luminescence Assay (PerkinElmer). 10^5 cells or 3*10^7 platelets were adjusted in 100 μl of cell suspension followed by the addition of 50 μl/well of mammalian cell lysis solution. The plate was left on orbital shaker for 5 minutes at 700 rpm. After Adding 50 μL/well of substrate solution, plate was shaken for another 5 minutes and incubated for 10 minutes at RT to stabilize the luminescent signal in the dark. Luminescence signals were collected on plate reader.

Murine model of FeCl3-induced carotid artery thrombosis

14-week-old *Jak2 V617F* mice were supplemented with regular water (n = 3) or 2% α-KG (n = 4) for 12 days. FeCl3-induced carotid artery thrombosis was induced as previously described (1). Mice were anesthetized, and the right common carotid artery was isolated and a flow probe (Transonic Systems, NY) was placed under the isolated carotid artery to monitor blood flow. Filter paper soaked with 5% FeCl3 solution was placed upon the carotid artery surface for 2.5 minutes,

followed by flushing with warmed saline to remove residue FeCl3 solution. Flow rate and vessel occlusion was monitored up to 30 minutes using Powerlab, and analyzed using LabChart Pro version 8.1 (ADInstruments, CO).

Murine model of carrageenan-induced thrombosis

10-week-old BALB/c mice were supplemented with regular water (n = 5) or 2% α-KG (n = 5) for 10 days. Carrageenan-induced thrombosis model was induced as previously described (2). Mice were injected with 100 µl of 10 mg/ml k-carrageenan (Sigma Aldrich) prepared in saline in intraperitoneal cavity. After 48 hours of carrageenan treatment, length and percentage of thrombus covered tail were measured and calculated.

Bulk RNA-seq analysis

RNA from mouse bone marrow and human purified CD14+ monocytes were extracted using RNeasy Mini Kit (Qiagen) with DNase treatment in duplicate. Samples were then indexed, pooled, and sequenced by Illumina NovaSeq 6000. TMM normalization size factors were calculated with EdgeR package (3.36.0) to adjust for samples for differences in library size. The TMM size factors and the matrix of counts were then use for differential expression analysis between conditions and the results were filtered for only those genes with adjusted p-values less than or equal to 0.05. GSEA was performed using software from Broad Institute (http://software.broadinstitute.org/gsea/index.jsp) as described previously (3). GSE2006 dataset was downloaded from GEO (https://www.ncbi.nlm.nih.gov/geo/).

scRNA-seq analysis

scRNA-Seq data were initially processed by the Cell Ranger pipeline (2.1.0) from 10 X Genomics using the human reference genome (GrCH38). The generated count matrices were then analyzed with the Seurat (4.3.0) package in R (4.1.3). Read counts were normalized to library size, scaled

by 10,000, log transformed, and filtered based on the following criteria: cells with less than 700 genes detected, proportion of the UMIs mapped to mitochondrial genes over 0.2 were excluded from analysis; Cell-cell variation in gene expression driven by the number of detected molecules and mitochondrial gene expression were regressed out using linear regression. Dimensionality reduction, PCA, UMAP projection, and graph-based clustering analysis were then performed. Marker genes for each cluster were identified with Wilcoxon rank sum test. Canonical markers were used to assign cell type identity to clusters (*GNLY* & *NKG7* for NK cells; *PPBP* & *PF4* for platelets; *CD79A* for B cells; *CD14* & *LYZ* for monocytes; *IL7R*, *S100A4* & *CCR7* for CD4+ T cells; *CD8A* for CD8+ T cells; *CD34* for HSCs). Seurat's AddModuleScore function was used to calculate module scores for feature expression programs in single cells. Gene sets for cell scoring were downloaded from MSIGDB (www.gsea-msigdb.org) (4, 5); lists of characteristic marker genes include platelet activation, signaling and aggregation (M1077), genes up-regulated in response to IFNG (M5913) and genes defining inflammatory response (M5932). Gene set enrichment analyses were run using GSEA software (4.2.3). GO and KEGG enrichment analyses were performed using clusterProfiler package (4.2.2) with differentially expressed genes (6, 7).
DoRothEA, a curated collection of transcription factors (TFs) and its transcriptional targets, and the statistical method VIPER were used to compute TF activities based on the mRNA expression levels of its targets (8, 9). Cells in scRNA-seq were re-clustered using TF activity computed with VIPER and DoRothEA. Top variable TFs in each type of cells were visualized for comparison.

Metabolomics

Metabolites were extracted from frozen platelet pellets at 4 x 10^8/mL by vigorous vortexing in the presence of ice cold 5:3:2 MeOH:MeCN:water (v/v/v) for 30 min at 4°C. Plasma samples were thawed on ice then a 20 µL aliquot was treated with 480 µL of the aforementioned solution then vortexed for 30 min at 4°C. Supernatants were clarified by centrifugation (10 min, 12,000 g, 4°C). The resulting extracts were analyzed (10 µL per injection for platelets, 20 µL per injection for

plasma) by ultra-high-pressure liquid chromatography coupled to mass spectrometry (UHPLC-MS - Vanquish and Q Exactive, Thermo). Metabolites were resolved on a Kinetex C18 column (2.1 x 150 mm, 1.7 µm) using a 5-minute gradient method exactly as previously described (10). Following data acquisition, .raw files were converted to .mzXML using RawConverter then metabolites assigned and peaks integrated using Maven (Princeton University) in conjunction with the KEGG database and an in-house standard library. Quality control was assessed as using technical replicates run at beginning, end, and middle of each sequence as previously described. Data were adjusted by protein quantity from proteomics. For data analysis, the peak intensities were normalized by median, log transformed and Pareto scaled on MetaboAnalyst website (www.metaboanalyst.ca). Processed data were analyzed with R using limma (3.50.1) package.

Proteomics

The samples were processed using the S-Trap filter (Protifi) according to the manufacturer's procedure. Briefly, a pellet after metabolomics extraction was solubilized in 50 µl of 5% SDS. Samples were reduced with 10 mM DTT at 55°C for 30 min, cooled to room temperature, and then alkylated with 25 mM iodoacetamide in the dark for 30 min. Next, a final concentration of 1.2% phosphoric acid and then six volumes of binding buffer (90% methanol; 100 mM triethylammonium bicarbonate, TEAB; pH 7.1) were added to each sample. After gentle mixing, the protein solution was loaded to a S-Trap filter, spun at 1,000 x g for 1 min, and the flow-through collected and reloaded onto the filter. This step was repeated three times, and then the filter was washed with 200 µL of binding buffer 3 times. Finally, 1 µg of sequencing-grade trypsin (Promega) and 150 µL of digestion buffer (50 mM TEAB) were added onto the filter and digested carried out at 37°C for 6 h. To elute peptides, three stepwise buffers were applied, with 100 µL of each with one more repeat, including 50 mM TEAB, 0.2% formic acid in H2O, and 50% acetonitrile and 0.2% formic acid in H2O. The peptide solutions were pooled, lyophilized and resuspended in 100 µl of 0.1% FA.

A 20 µl of each sample was loaded onto individual Evotips for desalting and then washed with 20 µL 0.1% FA followed by the addition of 100 µL storage solvent (0.1% FA) to keep the Evotips wet until analysis. The Evosep One system (Evosep) was used to separate peptides on a Pepsep column, (150 µm inter diameter, 15 cm) packed with ReproSil C18 1.9 µm, 120A resin. The system was coupled to the timsTOF Pro mass spectrometer (Bruker Daltonics) via the nano-electrospray ion source (Captive Spray, Bruker Daltonics).

The mass spectrometer was operated in PASEF mode. The ramp time was set to 100 ms and 10 PASEF MS/MS scans per topN acquisition cycle were acquired. MS and MS/MS spectra were recorded from *m/z* 100 to 1700. The ion mobility was scanned from 0.7 to 1.50 Vs/cm2. Precursors for data-dependent acquisition were isolated within ± 1 Th and fragmented with an ion mobility-dependent collision energy, which was linearly increased from 20 to 59 eV in positive mode. Low-abundance precursor ions with an intensity above a threshold of 500 counts but below a target value of 20000 counts were repeatedly scheduled and otherwise dynamically excluded for 0.4 min.

MS/MS spectra were extracted from raw data files and converted into .mgf files using MS Convert (ProteoWizard, Ver. 3.0). Peptide spectral matching was performed with Mascot (Ver. 2.6) against the Uniprot human database. Mass tolerances were +/- 15 ppm for parent ions, and +/- 0.4 Da for fragment ions. Trypsin specificity was used, allowing for 1 missed cleavage. Met oxidation, protein N-terminal acetylation and peptide N-terminal pyroglutamic acid formation were set as variable modifications with Cys carbamidomethylation set as a fixed modification.

Scaffold (version 5.0, Proteome Software) was used to validate MS/MS based peptide and protein identifications. Peptide identifications were accepted if they could be established at greater than 95.0% probability as specified by the Peptide Prophet algorithm. Protein identifications were accepted if they could be established at greater than 99.0% probability and contained at least two identified unique peptides. Data were analyzed with R using DEP package (1.16.0). Normalized data were input for gene set enrichment analysis.

Mass cytometry (CyTOF)

Whole blood was drawn into 4.5 mL tubes containing sodium heparin in accordance with an Institutional Review Board-approved protocol at Washington University in St. Louis. 200 μl whole blood were incubated with 200 μM Octyl-α-KG or DMSO for 1 hour followed by the stimulation of 10 ng/ml TNFα for 5 min. Add 1 x Proteomic Stabilizer from Smart Tube Inc (1.4ml of Stabilizer per ml of blood), mix gently and incubate for 10 minutes at room temperature. Keep samples at -80°C until it is ready to be stained. Frozen samples were thawed in cold water bath (10°C to 15°C) with agitation of the water for approximately 20 minutes until the samples are fully thawed. Samples were washed with Thaw-Lyse Buffer to lyse erythrocytes accordingly. Cells were then permeabilized, barcoded, stained with surface marker antibodies, permeabilized with methanol, stained with intracellular antibodies, and resuspended in DNA IR-intercalator. CyTOF experiments were conducted on a CyTOF2 mass cytometry (Fluidigm) with validated antibody panels. Post CyTOF run, cell identities were debarcoded and data was analyzed in Cytobank (cytobank.org). Monocytes were gated as follows: CD45+, CD66b-, CD14+. Raw median signal intensities were used for analysis.

References

1. Jones WL, Ramos CR, Banerjee A, Moore EE, Hansen KC, Coleman JR, et al. Apolipoprotein A-I, elevated in trauma patients, inhibits platelet activation and decreases clot strength. *Platelets.* 2022;33(8):1119-31.
2. Shrimali NM, Agarwal S, Kaur S, Bhattacharya S, Bhattacharyya S, Prchal JT, et al. alpha-Ketoglutarate Inhibits Thrombosis and Inflammation by Prolyl Hydroxylase-2 Mediated Inactivation of Phospho-Akt. *EBioMedicine.* 2021;73:103672.
3. Fisher DAC, Miner CA, Engle EK, Hu H, Collins TB, Zhou A, et al. Cytokine production in myelofibrosis exhibits differential responsiveness to JAK-STAT, MAP kinase, and NFkappaB signaling. *Leukemia.* 2019;33(8):1978-95.
4. Subramanian A, Tamayo P, Mootha VK, Mukherjee S, Ebert BL, Gillette MA, et al. Gene set enrichment analysis: a knowledge-based approach for interpreting genome-wide expression profiles. *Proc Natl Acad Sci U S A.* 2005;102(43):15545-50.
5. Liberzon A, Birger C, Thorvaldsdottir H, Ghandi M, Mesirov JP, and Tamayo P. The Molecular Signatures Database (MSigDB) hallmark gene set collection. *Cell Syst.* 2015;1(6):417-25.
6. Yu G, Wang LG, Han Y, and He QY. clusterProfiler: an R package for comparing biological themes among gene clusters. *OMICS.* 2012;16(5):284-7.
7. Wu T, Hu E, Xu S, Chen M, Guo P, Dai Z, et al. clusterProfiler 4.0: A universal enrichment tool for interpreting omics data. *Innovation (Camb).* 2021;2(3):100141.
8. Garcia-Alonso L, Holland CH, Ibrahim MM, Turei D, and Saez-Rodriguez J. Benchmark and integration of resources for the estimation of human transcription factor activities. *Genome Res.* 2019;29(8):1363-75.
9. Holland CH, Tanevski J, Perales-Paton J, Gleixner J, Kumar MP, Mereu E, et al. Robustness and applicability of transcription factor and pathway analysis tools on single-cell RNA-seq data. *Genome Biol.* 2020;21(1):36.
10. Nemkov T, Reisz JA, Gehrke S, Hansen KC, and D'Alessandro A. High-Throughput Metabolomics: Isocratic and Gradient Mass Spectrometry-Based Methods. *Methods Mol Biol.* 2019;1978:13-26.
