# Supplementary material for: Multiomic profiling reveals metabolic alterations mediating aberrant platelet activity and inflammation in myeloproliferative neoplasms
Source: J Clin Invest. 2024 Feb 1;134(3):e172256. doi: 10.1172/JCI172256 (PMC10836808; doi:10.1172/JCI172256)
Supplement: Supplemental table 2 [file jci-134-172256-s018.pdf]

Marker	Species specificity	Catalog/clone number	Manufacturer
PerCP-CD61	human	VI-PL2	Biolegend
FITC-CD41	human	HIP8	Biolegend
Pacific Blue- CD45	human	HI30	Biolegend
APC-CD62P	human	AK4	Biolegend
FITC-CD41/CD61	human	PAC-1	Biolegend
Tom20	human	F-10	Santa Cruz Biotechnology
β-Actin	human	13E5	Cell Signaling Technology
Total OXPHOS Cocktail	human	ab110411	Abcam
Akt	human	40D4	Cell Signaling Technology
Phospho-Akt (Ser473)	human	9271	Cell Signaling Technology
PLCbeta3	human	D9D6S	Cell Signaling Technology
Phospho-PLCbeta3 (Ser537)	human	D8K2R	Cell Signaling Technology
S6 Ribosomal Protein	human	54D2	Cell Signaling Technology
Phospho-S6 (Ser235/236)	human	2211	Cell Signaling Technology
HSP90 alpha/beta	human	F8	Santa Cruz Biotechnology
FITC-CD62P	mouse	Wug.E9	EMFRET Analytics
PE-CD41/CD61	mouse	JON/A	EMFRET Analytics
APC-CD61	mouse	2C9.G2	Biolegend
p44/42 MAPK (Erk1/2)	human	L34F12	Cell Signaling Technology
Phospho-p44/42(Thr202/Tyr204)	human	9101	Cell Signaling Technology
STAT3	human	10253-2-AP	Proteintech
Phospho-Stat3 (Tyr705)	human	9131	Cell Signaling Technology
Integrin β3	human	sc-365679	Santa Cruz Biotechnology
Alexa Fluor488-Integrin αIIb	human	sc-365938	Santa Cruz Biotechnology
Alexa Fluor594-α Tubulin	human	sc-5286	Santa Cruz Biotechnology
ME1	human	sc-100569	Santa Cruz Biotechnology
cathepsin C	human	sc-74590	Santa Cruz Biotechnology
Caspase-3	human	9662	Cell Signaling Technology
Cleaved Caspase-3 (Asp175)	human	9661	Cell Signaling Technology
Type III Collagen	human	1330-01	Southern Biotech
